# The *Arabidopsis* defensin gene, *AtPDF1*.*1*, mediates defence against *Pectobacterium carotovorum* subsp. *carotovorum* via an iron-withholding defence system

**DOI:** 10.1038/s41598-017-08497-7

**Published:** 2017-08-23

**Authors:** Pao-Yuan Hsiao, Chiu-Ping Cheng, Kah Wee Koh, Ming-Tsair Chan

**Affiliations:** 1 Institute of Plant Biology, National Taiwan University, Taipei, 10617 Taiwan; 2 Academia Sinica Biotechnology Center in Southern Taiwan, Tainan, 74145 Taiwan; 3 Agricultural Biotechnology Research Center, Academia Sinica, Taipei, 11529 Taiwan

## Abstract

Plant defensins (PDFs) are cysteine-rich peptides that have a range of biological functions, including defence against fungal pathogens. However, little is known about their role in defence against bacteria. In this study, we showed that the protein encoded by *ARABIDOPSIS THALIANA PLANT DEFENSIN TYPE 1*.*1* (*AtPDF1*.*1*) is a secreted protein that can chelate apoplastic iron. Transcripts of *AtPDF1*.*1* were induced in both systemic non-infected leaves of *Arabidopsis thaliana* plants and those infected with the necrotrophic bacterium *Pectobacterium carotovorum* subsp. *carotovorum* (*Pcc*). The expression levels of *AtPDF1*.*1* with correct subcellular localization in transgenic *A*. *thaliana* plants were positively correlated with tolerance to *Pcc*, suggesting its involvement in the defence against this bacterium. Expression analysis of genes associated with iron homeostasis/deficiency and hormone signalling indicated that the increased sequestration of iron by apoplastic AtPDF1.1 overexpression perturbs iron homeostasis in leaves and consequently activates an iron-deficiency-mediated response in roots via the ethylene signalling pathway. This in turn triggers ethylene-mediated signalling in systemic leaves, which is involved in suppressing the infection of necrotrophic pathogens. These findings provide new insight into the key functions of plant defensins in limiting the infection by the necrotrophic bacterium *Pcc* via an iron-deficiency-mediated defence response.

## Introduction

Organisms have evolved multiple defence strategies for protection against pathogen attack, many of which are triggered by environmental cues. One such strategy is the innate immune response, which utilizes Toll-like or pattern-recognition receptors to perceive specific pathogen-associated molecular patterns (PAMPs) that are derived from pathogens to trigger the defence signalling pathways. In plants, this process involves the activation of the salicylic acid (SA), jasmonic acid (JA) and ethylene (ET) signaling^1^ pathways and the biosynthesis of defence molecules, such as antimicrobial peptides (AMPs)^2, 3^, which cause membrane disruption, pore formation, and lead to cell lysis and death^4^. Based on sequence similarity, disulphide bond patterns and characteristic structural folds, AMPs can be categorized into several groups, including defensins, thionins, hevein-like peptides, knottin, α-hairpinin and lipid transfer proteins (LTPs)^5–7^.

Defensins constitute a well-studied group of relatively small AMPs (34–54 amino acids) with cysteine-rich motifs, which are present in a taxonomically broad range of multicellular eukaryotic organisms^8^. Unlike animal defensins which mainly possess anti-bacterial properties, plant defensins (PDFs), which have a highly conserved cysteine composition but are highly variable in their primary sequence^8^, have primarily been associated with protection against fungi^5, 8, 9^ and heavy metal stress^10, 11^. In contrast to the well-established actions of plant PDFs in limiting fungal infection, little is known about their roles and modes of action against bacteria^5^.

To date, more than 300 defensin-like (*DEFL*) genes have been identified in the model plant *Arabidopsis thaliana* ^12^. *AtPDF1*.*1* was originally described as encoding a seed-localized protein^13^, but its expression was later reported to be induced in leaves infected with the fungus *Blumeria graminis* f. sp. *hordei* ^14, 15^.

An association between PDFs and metal homeostasis was also indicated by our preliminary data, which showed that *AtPDF1*.*1* expression was induced upon iron overloading but not iron deficiency (Fig. S1B,D). Iron is essential for most living organisms and is involved in many biological processes^16^. In plants, intracellular iron is maintained at a very low level and excess iron is sequestered and stored in the apoplast and organelles, including the vacuole and plastid, to reduce iron toxicity^17^. Accordingly, iron homeostasis in plants is tightly controlled at the transcriptional and posttranslational levels^18, 19^. For example, Fe-deficiency-induced transcription factor 1 (FIT1) has been shown to interact with other iron homeostasis responsive transcription factors, such as basic helix-loop-helix 38 and 39 (bHLH38 and bHLH 39), to activate the expression of *FERRIC CHELATE REDUCTION OXIDASE 2* (*FRO2*) and *IRON-REGULATED TRANSPORTER 1* (*IRT1*) in *A*. *thaliana* ^20^.

Iron is also important for bacterial pathogens as it is required for their metabolism, enzymatic functions and pathogenesis. In order to acquire iron, plant pathogens utilize host iron-containing compounds such as heme^21^, transferrin, and lactoferrin, or synthesize siderophores to compete for iron uptake with their hosts^22^. Moreover, genes associated with immunity and iron homeostasis are also induced in response to treatment with the iron chelators ethylenediamine- di(o-hydroxyphenylacetic acid) (EDDHA) and desferrioxamine (DFO), suggesting that iron sequestration activates an immune response^23^. Together these findings demonstrated the existence of a relationship between perturbation of iron distribution and immunity in plants.

Given that AtPDF1.1 confers plant tolerance to fungi and that our preliminary data revealed increased transcript levels of *AtPDF1*.*1* following iron overloading, in this study, we provide evidence that apoplastic AtPDF1.1 perturbs iron homeostasis via iron chelation, resulting in activation of the iron-deficiency-induced ethylene signalling pathway. This response confers protection against infection by *Pectobacterium carotovorum* subsp. *carotovorum* (*Pcc*), a necrotrophic pathogen that causes bacterial soft rot (BSR) and serious production losses of a wide range of economically important crops worldwide^24^.

## Results

### Transcript levels of *AtPDF1s* increase in response to iron treatment and *Pcc* infection

The overexpression of *AhPDF1*.*1* in *A*. *thaliana* and yeast has been shown to enhance tolerance to zinc^10^. To evaluate whether *PDF1* genes in *A*. *thaliana* are similarly involved in zinc response, the expression profiles of the orthologs of *AhPDF1*.*1*, namely *AtPDF1*.*1*, *AtPDF1*.*2a* and *AtPDF1*.3, were monitored during ZnSO_4_ treatment. The results showed that the expression of *AtPDF1*.*2a* and *AtPDF1*.*3*, but not *AtPDF1*.*1*, was upregulated by ZnSO_4_ treatment (Fig. S1A), whereas transcript levels of these three *AtPDF1* genes were upregulated by treatment with other essential divalent ions, such as FeSO_4_ and CuSO_4_ (Fig. S1B,C). This suggested that *AtPDF1*.*1* may be involved in iron and copper homeostasis, but not in zinc homeostasis. In contrast to iron overloading, the expression of *AtPDF1* gene was not dramatically affected by iron deficient treatment (Fig. S1D).

To evaluate whether these three *AtPDF1* genes are involved in defence against bacterial pathogens, their transcriptional profiles were monitored in *A*. *thaliana* infected with *Pcc*. Time-course studies revealed that all three *AtPDF1* genes were upregulated at the infection site 1 hour post infection (hpi), with maximum expression occurring at 3 hpi for *AtPDF1*.*2* and *AtPDF1*.*3*, and at 4 hpi for *AtPDF1*.*1* (Fig. S2A). Analysis of non-inoculated leaves from infected plants revealed that the transcript levels of *AtPDF1*.*1*, but not *AtPDF1*.*2* or *AtPDF1*.*3*, were induced systemically at 24 hpi (Fig. S2B). In order to investigate the protein level of AtPDF1.1, the AtPDF1.1:green fluorescent protein fusion protein (AtPDF1.1:GFP) under the control of the *AtPDF1*.*1* promoter was used to distinguish from other native defensins. When transgenic *A*. *thaliana* plants expressing AtPDF1.1:GFP were infected with *Pcc*, maximum protein accumulation was observed at the local site of infection at 3–4 hpi (Fig. S2C). Furthermore, in addition to a protein band of ~36 kDa, which corresponds to the predicted size of the AtPDF1.1-GFP fusion protein, a protein of ~33 kDa was also dramatically accumulated at the local site of infection at 5 hpi and in systemic leaves at 24 hpi (Fig. S2C), suggesting that AtPDF1.1 may have undergone post-translational modification, and *AtPDF1*.*1* expression is induced both locally and systemically upon *Pcc* infection. Along with this notion, analysis of the predicted AtPDF1.1 protein sequence using SignalP 4.1 software (http://www.cbs.dtu.dk/services/SignalP/) revealed the presence of a putative N-terminal secretory signal peptide (Fig. S3A), suggesting that the AtPDF1.1 protein may be targeted to the secretory pathway^25, 26^, and the 33-kDa protein is likely resulted from the cleavage of the N-terminal signal. Consistent with this targeting prediction, the first identified plant defensin was reported to be a secreted protein released from germinated radish seeds^27^.

### *AtPDF1*.*1* confers plant tolerance to *Pcc* infection

**Figure 1:**
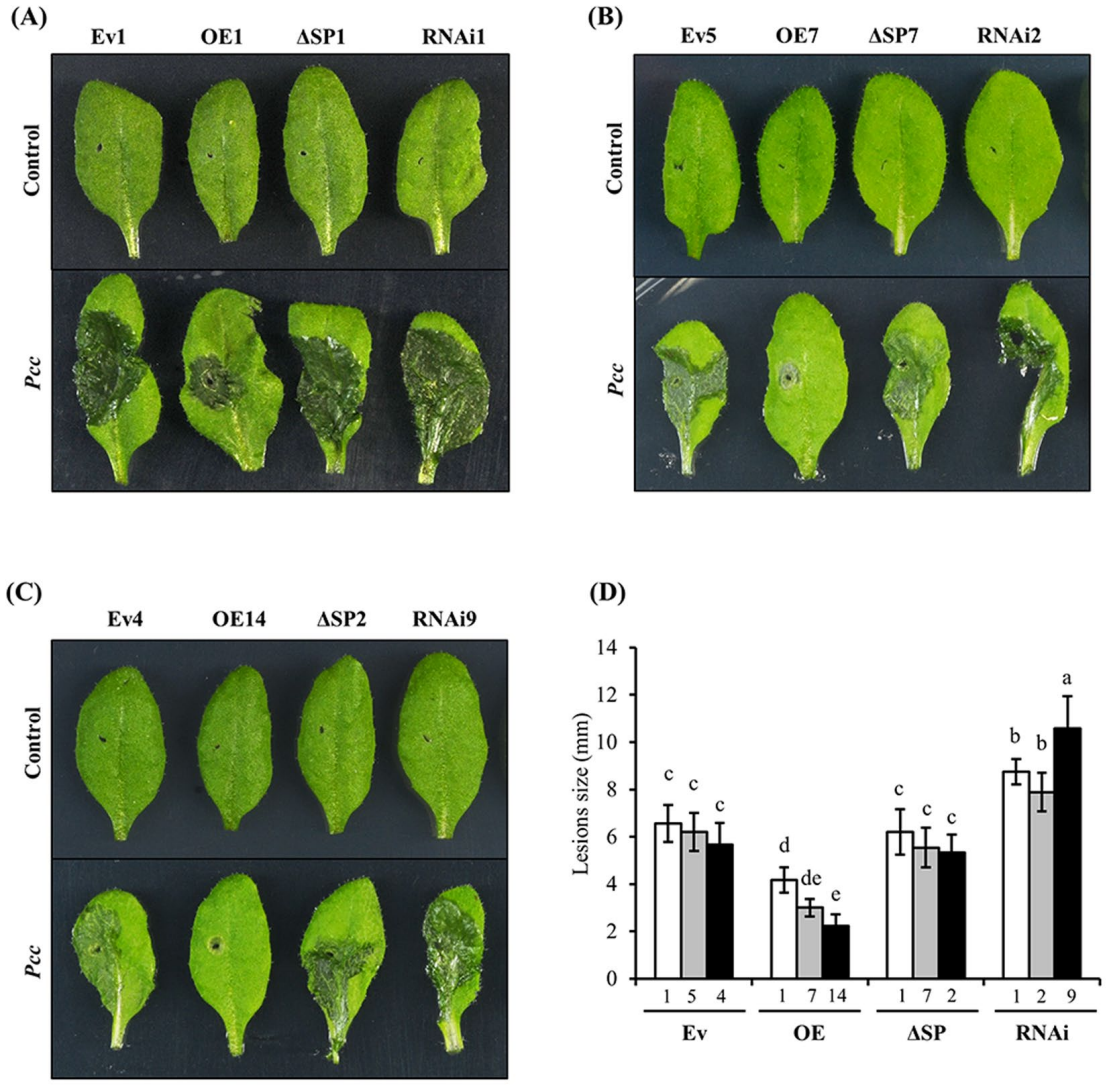
Expression levels of *AtPDF1*.*1* correlate with protection against *Pcc* infection. (**A**,**B** and **C**) Transgenic *Arabidopsis thaliana* plants (Ev: line1, 5, 4; OE: line1, 7, 14; ΔSP: line1, 7, 2; RNAi: line1, 2, 9) were inoculated with *Pcc* or water (control) and *Pcc*-mediated symptoms were evaluated at 24 hpi. (**D**) The maximal lesion sizes (diameter; mm) on leaves, denoting bacterial soft rot severity, were determined at 24 hpi. Values are means ± standard errors from ten samples for each line and each infection in a single experiment that was repeated at least three times with similar results. Different letters above each bar indicate significant difference (Fisher’s Least Significant Difference (LSD) LSD post hoc one-way ANOVA, *P* < 0.05).

Since *AtPDF1*.*1* transcript levels increased in response to *Pcc* infection (Fig. S2), we then investigated the role of *AtPDF1*.*1* in defending against pathogenic bacteria. Suppression (RNAi) transgenic lines were generated, because *AtPDF1*.*1* expression in the only available *AtPDF1*.*1* T-DNA insertion mutant (SALK_029051) is unaltered compared to the wild-type plant. In addition, to determine whether the cellular localization of AtPDF1.1 is critical for its functions, transgenic plants overexpressing a truncated form of AtPDF1.1, lacking the N-terminal signal peptide (*AtPDF1*.*1*ΔSP) and *AtPDF1*.*1* overexpression (OE) were also generated (Fig. S3B). Quantitative PCR (qPCR) analysis verified the success of *AtPDF1*.*1* OE and RNAi in transgenic plants (Fig. S3C). The results showed that, compared with the empty vector (Ev) harbouring control plants, the *AtPDF1*.*1* OE plants exhibited enhanced tolerance to *Pcc* infection, the *AtPDF1*.*1*ΔSP plants exhibited similar disease response, while the *AtPDF1*.*1* RNAi plants exhibited increased susceptibility (Fig. 1). These results indicated that the tolerance to *Pcc* infection correlates well with the expression level of *AtPDF1*.*1*, and that the putative N-terminal secretory signal peptide, which could be a key determinant for the apoplastic location of AtPDF1.1, is crucial for its function in defence against *Pcc* infection.

### AtPDF1.1 is secreted into the apoplast

**Figure 2:**
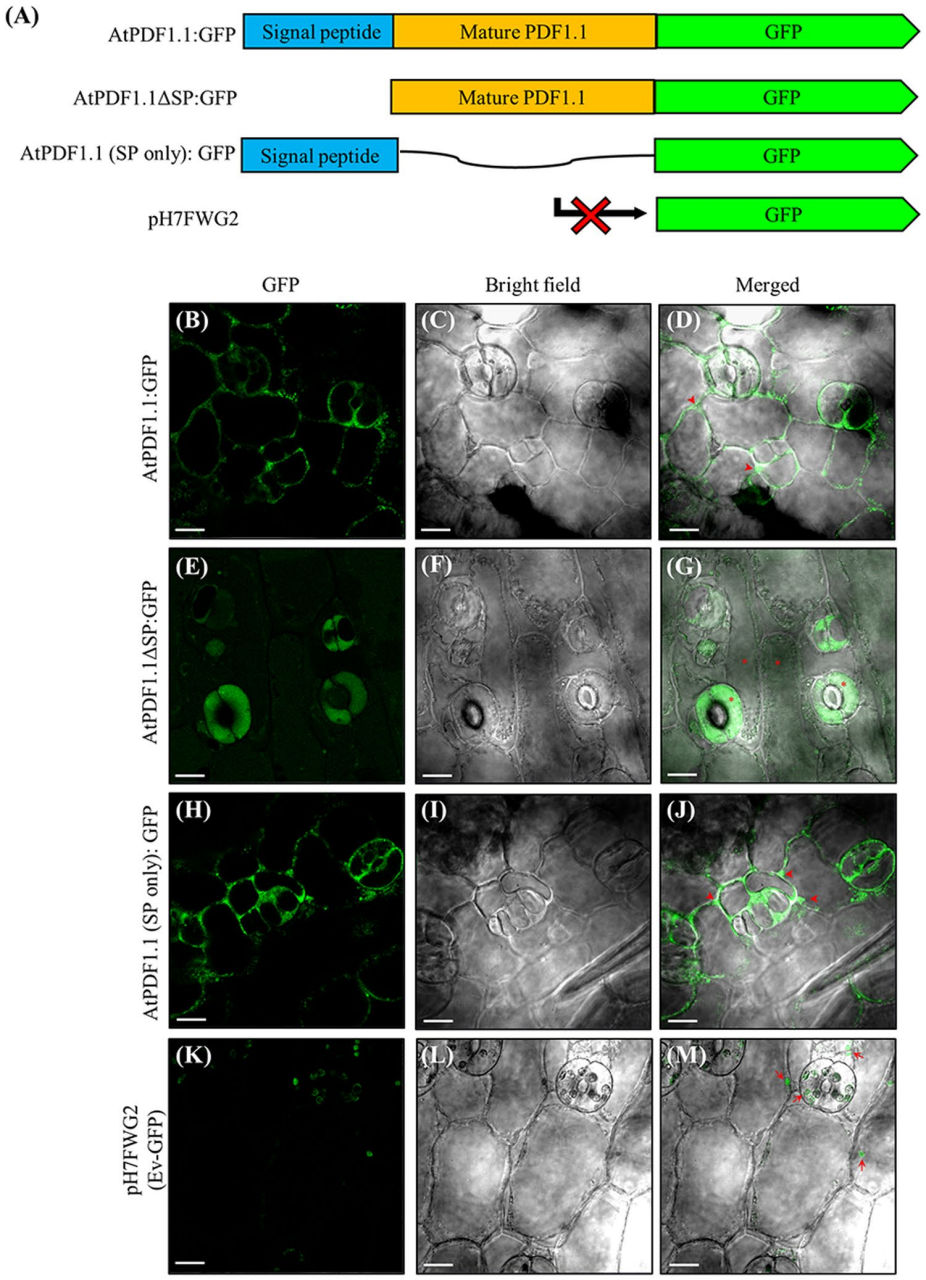
AtPDF1.1 fusion proteins are localized and accumulated in the apoplast. (**A**) Schematic diagram of the AtPDF1.1:GFP (green fluorescent protein), AtPDF1.1ΔSP:GFP and AtPDF1.1(SP only):GFP fusion constructs. The leaf epidermis of *AtPDF1*.*1:GFP* (**B**–**D**), *AtPDF1*.*1ΔSP:GFP* (**E**–**G**), *AtPDF1*.*1*(*SP only*)*:GFP* (**H**–**J**) and *Ev-GFP* transgenic *Arabidopsis thaliana* plants (**K**–**M**) were subjected to confocal microscopy to determine the subcellular localization of AtPDF1.1 fusion proteins. Left panel, GFP fluorescence; middle panel, bright field images; right panel, merged images. Each line was repeated at least three times with similar results. Asterisks (*) denote GFP signals detected in the cytosol (asterisks), arrowheads denote GFP signals detected in the apoplastic space and arrows denote the chlorophyll fluorescence. Bars, 10 μm.

To determine the subcellular localization of AtPDF1.1, we generated transgenic *A*. *thaliana* plants overexpressing *GFP* fused to the C-terminal of full-length *AtPDF1*.*1*-encoding gene (*AtPDF1*.*1:GFP*), or a truncated *AtPDF1*.*1*-encoding gene lacking either the signal peptide (*AtPDF1*.*1ΔSP:GFP*) or the mature protein region (*AtPDF1*.*1(SP only):GFP*) (Fig. 2A). *A*. *thaliana* lines harbouring the empty expression vector pH7FWG2 (Ev-GFP) were used as a negative control (Fig. 2A). Due to the presence of the *ccdB* and chloramphenicol-resistance (Cm^R^) gene cassette upstream of the GFP-encoding gene in this vector, *GFP* transcripts are not translated into functional protein. Confocal microscope imaging of the plasmolysed epidermis of the leaves of transgenic plants revealed that the GFP signal was localized in the apoplastic region surrounding the plasma membrane of *AtPDF1*.*1:GFP* and *AtPDF1*.*1(SP only):GFP* plants (Fig. 2B–D and H–J), while *AtPDF1*.*1ΔSP:GFP* plants showed the GFP signal in the cytosol (Fig. 2E–G). We concluded that the mature form of AtPDF1.1 is localized in the apoplast, which is consistent with the localization of other plant defensins^27, 28^.

To confirm that AtPDF1.1 is secreted, levels of the recombinant fusion protein were measured in the growth medium of suspension cells generated from transgenic plants (Fig. S4A) together with levels in protein lysates from whole seedlings, by western blot analysis with an anti-GFP antibody. Two GFP fusion protein bands were detected in the seedling lysates of *AtPDF1*.*1:GFP* and *AtPDF1*.*1(SP only):GFP* plants, suggesting that AtPDF1.1 with and without the N-terminal signal peptide were expressed and that AtPDF1.1(SP only):GFP (band 3 and 4) may increase molecular weight via certain post-translational modification (Fig. S4B). In contrast, a single GFP fusion protein band was observed in the protein lysate of *AtPDF1*.*1ΔSP:GFP* seedlings. When the proteins in the media of the suspension cells were analysed, only GFP fusion proteins containing the N-terminal signal peptide were detected in the supernatants of *AtPDF1*.*1:GFP* and *AtPDF1*.*1(SP only):GFP* suspension cell cultures, and no immunoreactive proteins were detected in the growth medium of *AtPDF1*.*1ΔSP:GFP* suspension cells (Fig. S4C). These data further suggest that the N-terminal signal peptide is responsible for targeting AtPDF1.1 to the apoplast. Because the apoplast is the initial site for *Pcc* infection^29^, and the correct localization of AtPDF1.1 is critical for its function in defence against *Pcc* infection (Fig. 1), our results are consistent with the idea that apoplastic AtPDF1.1 can interfere with *Pcc* infection.

### AtPDF1.1 localized to the apoplast is involved in iron distribution

The apoplast is a primary infection site for bacterial pathogens of plants, and pathogens such as *P*. *syringae* rely on extracellular nutrients^30^. The iron status of *A*. *thaliana* has been reported to be critical for the pathogenicity of the necrotrophic bacterium *Dickeya dadantii* ^31^. Under host iron limiting conditions, bacteria produce siderophores to enhance their iron uptake^32^. Since *AtPDF1*.*1* expression is responsive to both iron overloading (Fig. S1B) and *Pcc* infection (Fig. S2), we hypothesized that AtPDF1.1 may function as an iron chelator to limit the availability of iron for *Pcc* as part of an iron-withholding defence system^33^. To determine whether apoplastic iron affects *Pcc* pathogenesis, exogenous iron was supplemented to plants and disease response was assessed. The results showed that extra iron supplement to the leaves increased the susceptibility of all transgenic plants to *Pcc* invasion (Fig. S5), supporting the importance of apoplastic iron for *Pcc* infection. However, even with extra iron supplement, the *AtPDF1*.*1* OE plants still displayed the lowest susceptibility to *Pcc* infection, further supporting a positive role of AtPDF1.1 in plant defence against *Pcc*.

To evaluate whether AtPDF1.1 is involved in chelating/sequestering iron, we first determined whether it has the capacity to bind metal ions. In a metal affinity assay (Fig. S6), AtPDF1.1ΔSP showed binding affinity to iron ions. Since the resins derivatized from iminodiacetic acid exhibited different degrees of affinity to metal ions, with a preference for transition metals, such as cobalt (Co), nickel (Ni), copper (Cu) and zinc (Zn)^34–36^, we deduced that fewer Fe^3+^-conjugated resins are available for AtPDF1.1ΔSP protein to bind. This may explain the relatively weak protein band detected, and so the intensity of detection signal does not correlate with the binding affinity between the metal ions and AtPDF1.1ΔSP protein. Overall, the data suggest that AtPDF1.1ΔSP protein is able to bind to iron ions.

**Figure 3:**
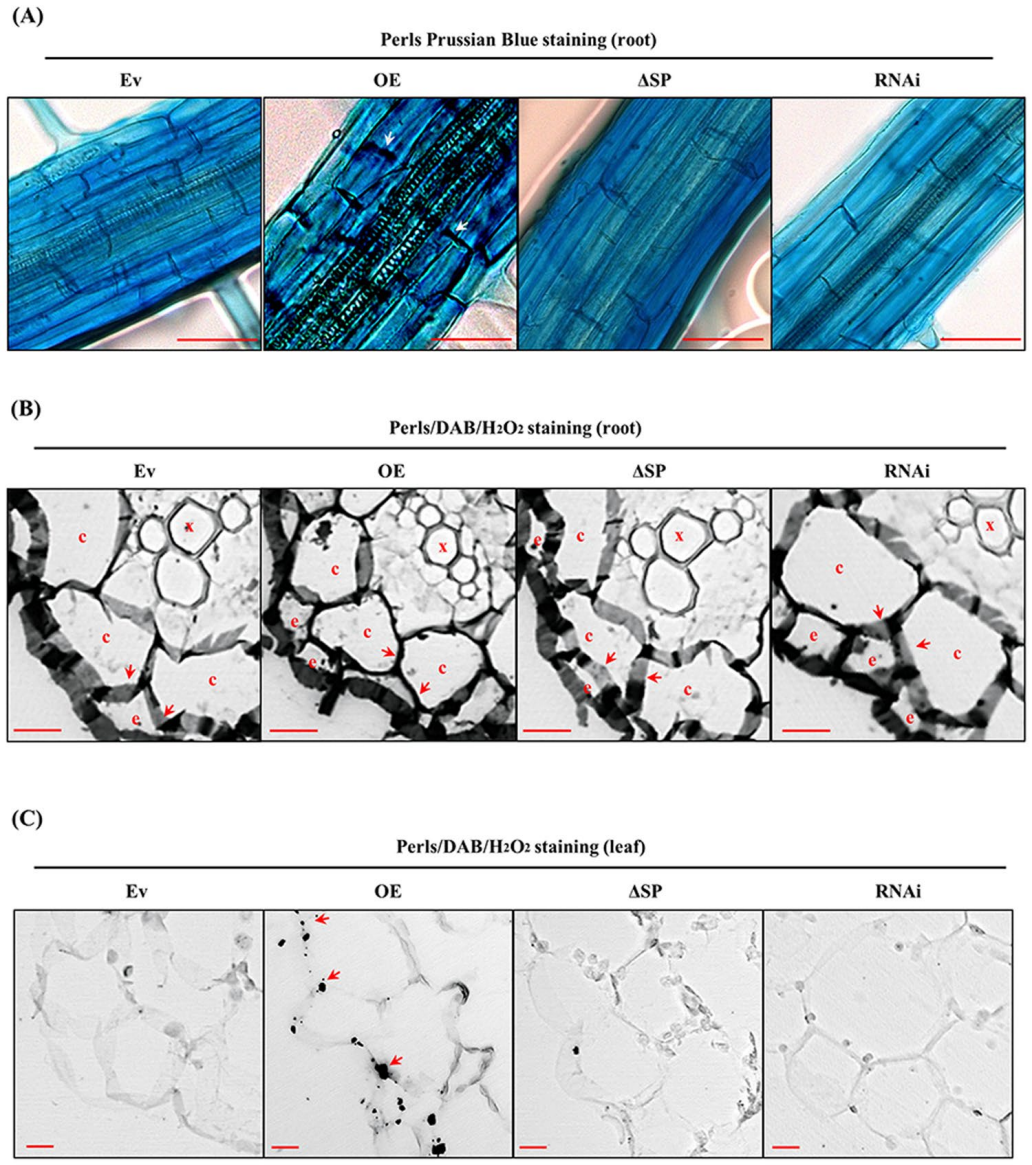
Iron aggregate is accumulated in the apoplast by AtPDF1.1 protein. The roots of four-week-old hydroponically grown transgenic *A*. *thaliana* plants were harvested. (**A**) The root epidermis was subjected to Perls Prussian Blue staining, and iron distribution was examined. Bars, 50 μm. Transverse sections of roots (**B**) and leaves (**C**) were subjected to Perls/DAB/H_2_O_2_ staining and iron distribution was examined. Each line was repeated at least three times with similar results. Arrows indicate the presence of iron accumulating in the apoplast; e, c and x denote the epidermis, cortex and xylem, respectively. Bars, 10 μm.

To further evaluate the iron binding affinity of the AtPDF1.1 protein *in planta*, transgenic plants were grown under hydroponic conditions and their iron acquisition capabilities were compared. As shown in Fig. 3A, Perls staining revealed a high abundance of iron ions in the intercellular regions of the root epidermis of *AtPDF1*.*1* OE plants, but only weak staining was observed in the Ev, *AtPDF1*.*1*ΔSP and *AtPDF1*.*1* RNAi transgenic plants. Furthermore, sections of roots and leaves from transgenic plants were subjected to Perls/diaminobenzidine (DAB)/H_2_O_2_ staining and then examined by confocal microscopy to spot regions with iron deposits. Consistent with previous studies of *A*. *thaliana* wild-type plants^37^, the apoplastic regions of the cortex of roots were stained in all sections (Fig. 3B), indicating extracellular iron accumulation. However, compared to the Ev, *AtPDF1*.*1ΔSP* and *AtPDF1*.*1RNAi* transgenic plants, more heavily stained zones were observed in the apoplast of the root epidermis and cortex of *AtPDF1*.*1 OE* plants. Similarly, substantial amounts of iron aggregates were observed in the leaf apoplast of *AtPDF1*.*1* OE plants (Fig. 3C), while only low levels of iron aggregates were present in the Ev, *AtPDF1*.*1*ΔSP and *AtPDF1*.*1* RNAi plants. Since the amounts of iron aggregates accumulated in the apoplast, where AtPDF1.1 is localized (Fig. 2), correlated well with the expression levels of AtPDF1.1 (Fig. 3) and AtPDF1.1 can bind to iron (Fig. S6), we projected that AtPDF1.1 can chelate/sequester iron.

We next determined whether apoplastic sequestration of iron by AtPDF1.1 might affect the intracellular iron content. The results showed that *AtPDF1*.*1* OE and *AtPDF1*.*1* RNAi plants, respectively, had the highest and lowest apoplastic iron content in the roots and leaves (Fig. S7A,B), and that the levels of apoplastic iron were inversely correlated with intracellular iron content (Fig. S7C,D). Given that roots are the site for iron uptake^38^, we concluded that the binding of iron by AtPDF1.1 in the root apoplast might limit the amount of intracellular iron, hence accounting for the low levels of iron in the root and leaves of the *AtPDF1*.*1* OE plants.

We next evaluated the effect of *Pcc* infection on systemic iron content. As shown in Fig. S7A and C, *Pcc* infection caused a decrease in root apoplastic iron content and an increase in root intracellular iron content. In contrast, an increase in leaf apoplastic iron content after *Pcc* infection was observed in all transgenic plants, except the *AtPDF1*.*1* OE lines (Fig. S7B), while the leaf intracellular iron content was not significantly changed (Fig. S7D). *AtPDF1*.*1* overexpression seemed to restrict iron in leaf apoplasts without significantly affecting the leaf intracellular iron content. Because *Pcc* infection is mainly confined to leaves during the duration of our experiments, these results suggested that *Pcc* infection results in root apoplastic iron depletion and induces a change in root-to-shoot translocation of iron.

When transgenic plants were grown under long-term-hydroponic conditions, slight chlorosis was observed in the leaves of the *AtPDF1*.*1* OE plants, but chlorophyll content was higher and leaves were greener in the *AtPDF1*.*1* RNAi plants (Fig. S8). Iron is involved in the formation of δ-aminolevulinic acid, which is required for chlorophyll formation, and loss of chlorophyll is associated with a lack of iron availability^39^. Since the iron distribution of the leaf was aggregated in the apoplast of *AtPDF1*.*1* transgenic OE plants (Figs 3C and 7B), these results are consistent with the observation that the *AtPDF1*.*1* OE plant had lower chlorophyll content than the others (Fig. S8).

### Chelation of iron in the leaf by apoplastic AtPDF1.1 induces an iron deficiency response

**Figure 4:**
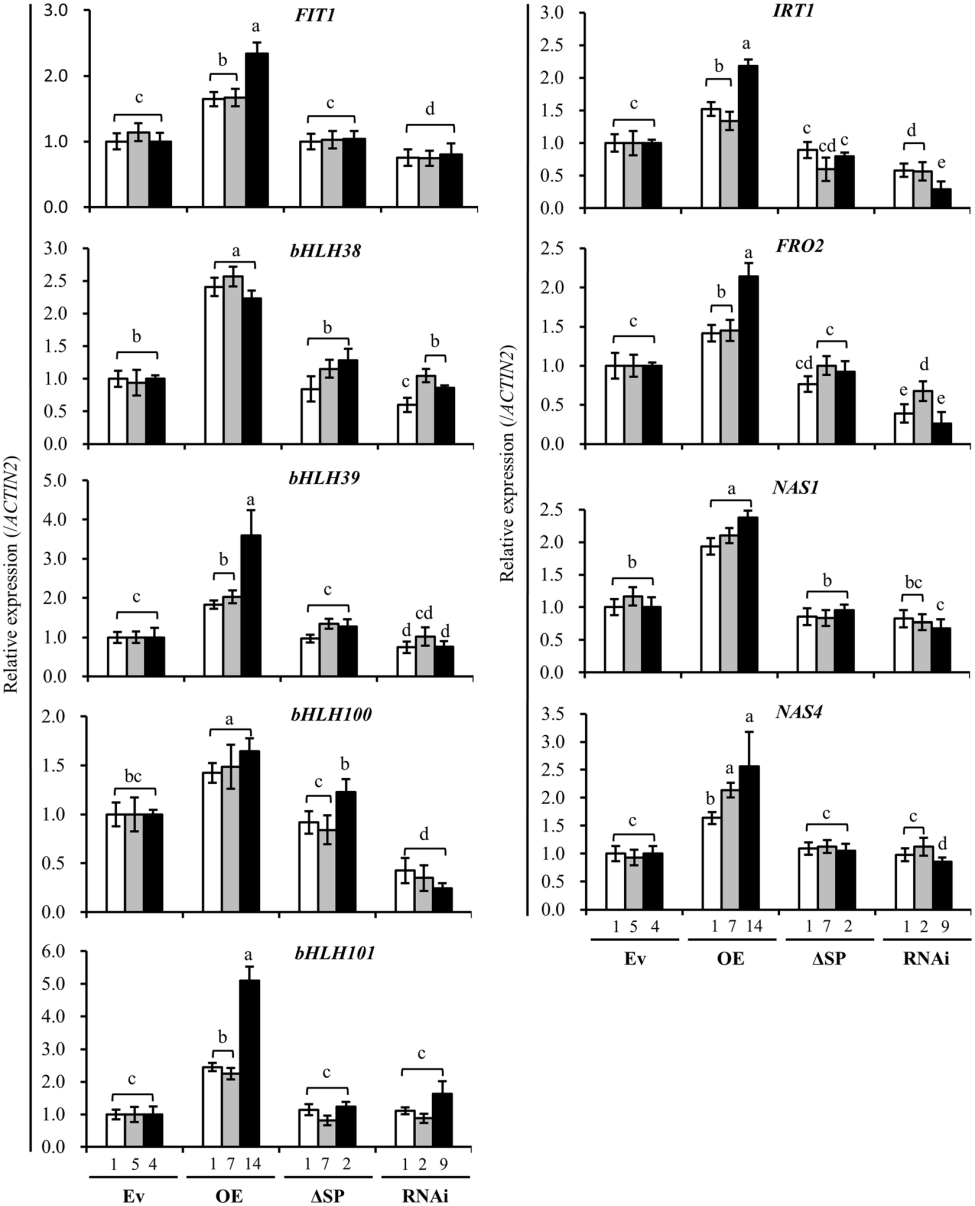
Iron deficiency-associated genes are upregulated in *AtPDF1*.*1* OE plants. The leaves and roots were harvested from hydroponically grown transgenic *A*. *thaliana* plants (Ev: line 1, 5, 4; OE: line1, 7, 14; ΔSP: line 1, 7, 2; RNAi: line 1, 2, 9) and the expression levels of genes associated with iron homeostasis (*FIT1*, *bHLH38*, *bHLH39*, *bHLH100* and *bHLH101*) in the leaves, and iron deficiency response (*IRT1*, *FRO*2, *NAS1 and NAS4*) in the roots were monitored by qPCR and compared with expression in the empty vector control plants (Ev4, defined value of 1). Transcript levels were normalized to those of *ACTIN2*. Values are means ± standard errors from twelve samples for each line in a single experiment that was repeated at least three times with similar results. Different letters above each bar indicate significant difference (LSD post hoc one-way ANOVA, *P* < 0.05).

Given that the apoplastic iron content is positively associated with *AtPDF1*.*1* expression level, we next examined whether the activation of the regulatory pathways involved in iron homeostasis correlated with *AtPDF1*.*1* expression. Analysis of the transcriptional profiles of the iron homeostasis transcription factor genes, *FIT1*, *bHLH38*, bHLH39, *bHLH100* and *bHLH101* ^40^ showed that, under non-infected condition, these genes were significantly upregulated in the leaves of *AtPDF1*.*1* OE plants, and, with the exception of *FIT1*, *bHLH38* and *bHLH101*, were downregulated in the *AtPDF1*.*1* RNAi plants, (Fig. 4). Similarly, transcript levels of the iron-deficiency-responsive genes, *IRT1*, *FRO2*, *NAS1* and *NAS4* ^41, 42^ were higher and lower, respectively, in the roots of *AtPDF1*.*1* OE and *AtPDF1*.*1* RNAi plants compared with the Ev plants (Fig. 4). This suggests that the high levels of AtPDF1.1 protein present in the apoplast result in a perturbation of iron homeostasis and consequently a state of intracellular iron deficiency.

When plants were subjected to *Pcc* infection, transcript levels of the iron homeostasis transcription factor genes, *bHLH38*, *bHLH39* and *bHLH100* and the iron-deficiency-responsive genes, *IRT1*, *FRO2*, and *NAS4*, were increased in the systemic leaves or roots of all transgenic plants (Fig. S9). This indicated that *Pcc* infection induces an iron deficiency response that is independent of *AtPDF1*.*1* transcript levels.

Ferric chelate reductase (FCR) is required to reduce Fe^3+^ to Fe^2+^ in the rhizoshere to allow iron uptake into the cell, and its activity is highly activated in response to iron deficiency^43^. As shown in Fig. S10, FCR activity was the highest and lowest, respectively, in the roots of *AtPDF1*.*1* OE and *AtPDF1*.*1* RNAi plants, suggesting that the *AtPDF1*.*1* OE plants were experiencing greater iron deficiency. In addition, FCR activity was further enhanced by *Pcc* infection, further confirming that *Pcc* infection can lead to an iron deficiency response (Fig. S10). The observation that *Pcc* infection caused an increase in FCR activity in all transgenic plants was consistent with the decreased root apoplastic iron content and the increased root intracellular iron content, respectively (Fig. S7A,C).

### A defence response is activated upon the induction of an iron deficiency response

To examine whether iron aggregation in the apoplast increased as a result of *Pcc* infection, both locally infected and systemic leaves were harvested at 4 and 24 hpi and subjected to Perls/DAB/H_2_O_2_ staining analysis. In the absence of *Pcc* infection, iron aggregates were only detected in the leaves of the *AtPDF1*.*1* OE plants. When plants were infected with *Pcc*, enhanced accumulation of iron aggregates was observed in the local and systemic leaf tissues of all transgenic plants, particularly near to the vascular bundles (Fig. S11). This suggested that *Pcc* infection induced iron accumulation both locally and systemically, and that the accumulation of iron aggregates was further enhanced by the presence of AtPDF1.1. AtPDF1.1-mediated enhancement on *Pcc*-induced iron accumulation would account for the overall perturbation of iron homeostasis and elicitation of the iron deficiency response observed in transgenic lines (Figs S9 and S10).

**Figure 5:**
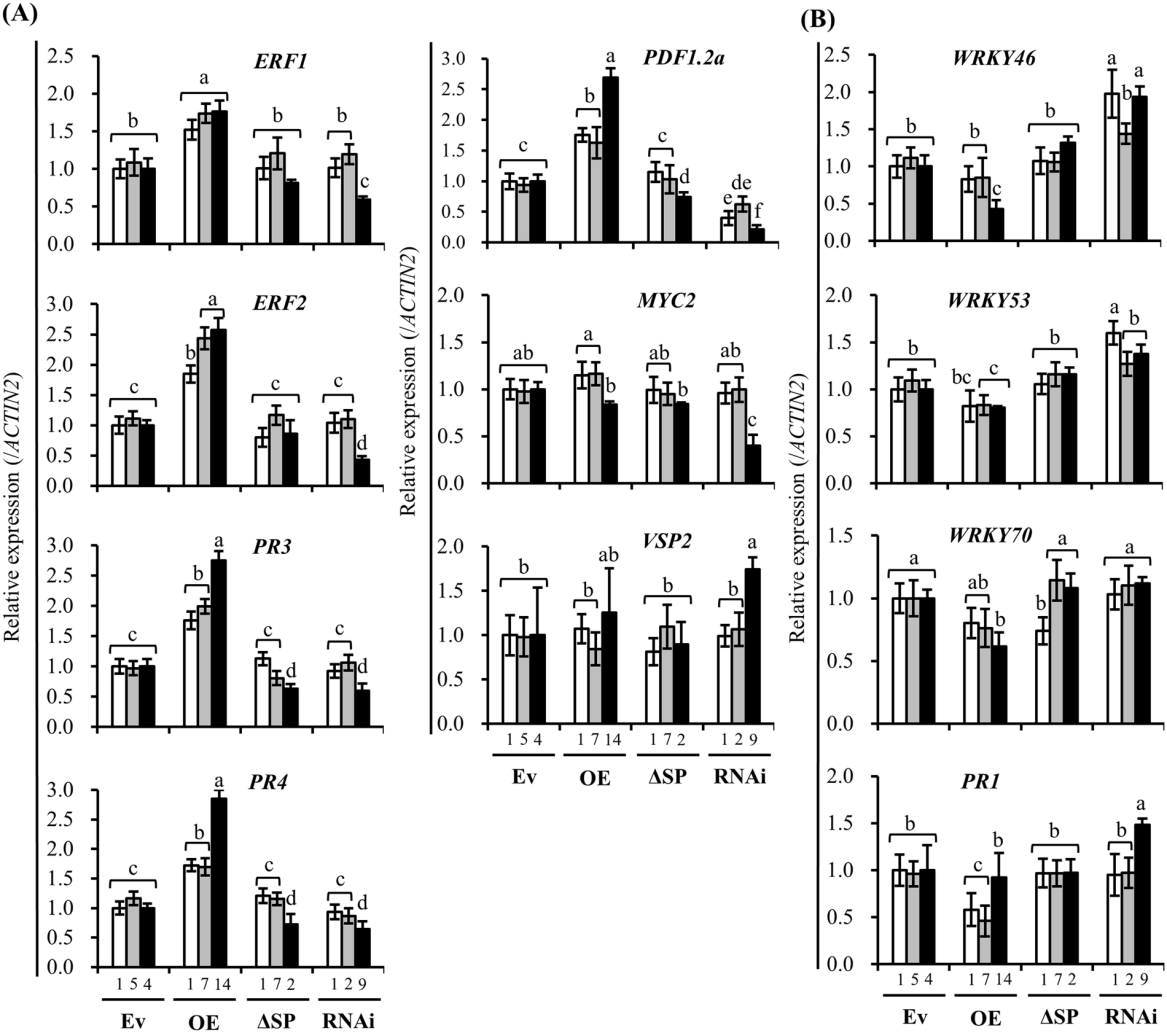
Expression levels of downstream genes of *ERF1*/*2* are increased in *AtPDF1*.*1* OE plants. The leaves were harvested from hydroponically grown transgenic *A*. *thaliana* plants (Ev: line 1, 5, 4; OE: line 1, 7, 14; ΔSP: line 1, 7, 2; RNAi: line 1, 2, 9) and the expression levels of JA/ET-responsive genes (*ERF1*, *ERF2*, *PR3*, *PR4*, *PDF1*.*2a*, *MYC2* and *VSP2*; (**A**)) and SA-responsive genes (*WRKY46*, *WRKY53*, *WRKY70* and *PR1*; (**B**)) were monitored by qPCR and compared with expression in empty vector control plants (Ev4, defined value of 1). Transcript levels were normalized to those of *ACTIN2*. Values are means ± standard errors from twelve samples for each line in a single experiment that was repeated at least three times with similar results. Different letters above each bar indicate significant difference (LSD post hoc one-way ANOVA, *P* < 0.05).

Studies by Alia Dellagi *et al*.^33^ revealed that the sequestration of iron by synthetic iron chelators or bacterial siderophores can induce the expression of genes associated with iron homeostasis and the subsequent activation of immune responses, including callose deposition, hydrogen peroxide production and a SA-mediated response^23^. The transcriptional profiles of JA/ET- and SA-responsive genes were evaluated to investigate whether the AtPDF1.1-mediated activation of iron homeostasis genes (Fig. 4) also triggered a plant defence response^23^. As shown in Fig. 5A, expression of the JA/ET-responsive genes, *ERF1*, *ERF2*, *PR3*, *PR4* and *AtPDF1*.*2a*, was upregulated in the *AtPDF1*.*1* OE plants. However, the expression of *VSP*2 *and MYC*2, which are in the MYC-branch of the JA response pathway^44^, was not induced in any of transgenic plants. This suggests that ET signalling, but not JA signalling, may respond to altered *AtPDF1*.*1* expression. In contrast, most of the tested genes associated with the SA signalling pathway (*WRKY46*, *WRKY*5*3* and *WRKY70* and *PR1*) were vaguely downregulated and upregulated, respectively, in the *AtPDF1*.*1* OE and *AtPDF1*.*1 RNAi* plants (Fig. 5B). Upon *Pcc* infection, genes associated with the JA/ET responses were upregulated in all transgenic plants, with the highest induced levels in the *AtPDF1*.*1* OE plants (Fig. S12A). In contrast, the expression profiles of genes associated with the SA response were inconsistent and not correlate with *AtPDF1*.*1* expression levels (Fig. S12B). These results suggest that AtPDF1.1 action and *Pcc* infection promote the JA-/ET-mediated signalling. Since the JA/ET signalling pathway is associated with defence against necrotrophic pathogens, including *Pcc* ^45^, these results may explain the enhanced tolerance to *Pcc* infection in the *AtPDF1*.*1* OE plants (Fig. 1), but not to the hemibiotrophic bacterium *Pst* DC3000 (Fig. S13).

**Figure 6:**
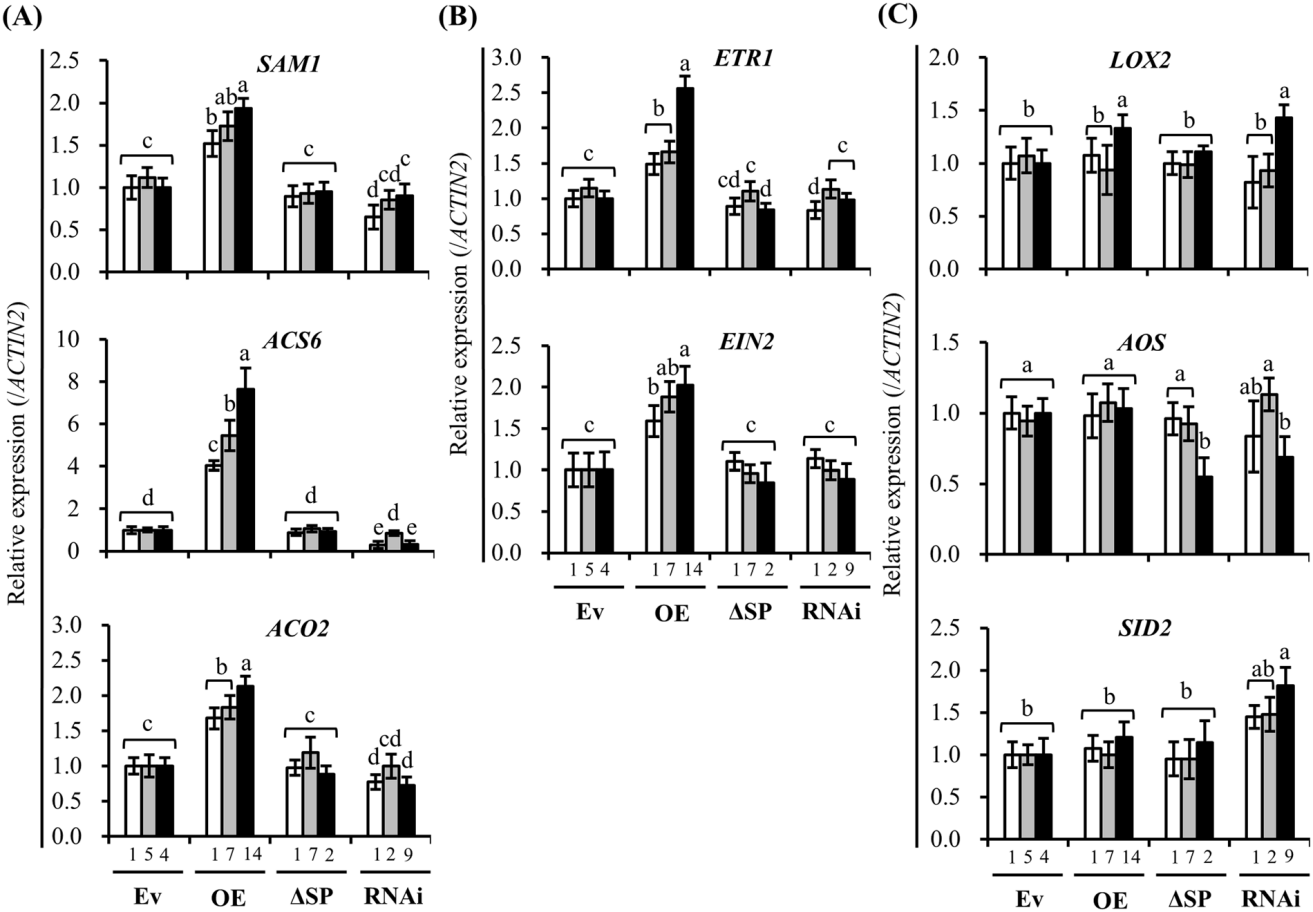
ET-signalling genes are upregulated in *AtPDF1*.*1* OE plant. The roots from hydroponically-grown transgenic plants (Ev: line1, 5, 4; OE: line1, 7, 14; ΔSP: line1, 7, 2; RNAi: line1, 2, 9) were harvested and transcript levels of ET-biosynthesis genes (*SAM1*, *ACS6* and *ACO2*; (**A**)), ET-signalling genes (*ETR1* and *EIN2*; (**B**)) JA-/SA-biosynthesis genes (*LOX2*, *AOS* and *SID2*; (**C**)) were monitored by qPCR and compared with expression in empty vector control plants (Ev4, defined value of 1). Transcript levels were normalized to *ACTIN2*. Values are means ± standard errors from twelve samples for each line in a single experiment that was repeated at least three times with similar results. Different letters above each bar indicate significant difference (LSD post hoc one-way ANOVA, *P* < 0.05).

The state of iron deficiency leads to the induction of ethylene biosynthesis in the roots^46, 47^. Since iron deficiency response occurred in the leaves and roots of *AtPDF1*.*1* OE plants (Figs 4 and S10), we evaluated whether the state of iron deficiency induced by AtPDF1.1 overexpression also led to activation of ethylene signalling in the roots. As shown in Fig. 6, the ET-biosynthesis genes, *SAM1*, *ACS6* and *ACO*2 (Fig. 6A) and ET-signalling genes, *ETR1* and *EIN*2 (Fig. 6B) were expressed at higher levels in *APDF1*.*1* OE plants compared to the other transgenic plants. The activation of ET signalling in the *AtPDF1*.*1* OE plants may explain why the JA-/ET-responsive *ERF1*/*2* and their downstream genes, but not the JA-responsive *MYC2* and *VSP2*, were upregulated (Fig. 5A)^48^. Furthermore, the expression of JA/SA biosynthesis genes was not significantly affected by *AtPDF1*.*1* (Fig. 6C), but the expression of ET/JA biosynthesis genes and ET signalling genes in the roots was increased at varied levels after *Pcc* infection (Fig. S14). To further verify whether ethylene signalling is involved in AtPDF1.1-mediated tolerance to *Pcc*, the ethylene inhibitor 1-methylcyclopropene (1-MCP) was applied to transgenic plants, followed by inoculation with *Pcc*. With the 1-MCP treatment, the soft rot symptom caused by *Pcc* was enhanced in all of transgenic plants, including the *AtPDF1*.*1* OE plants (Fig. S15). This result further points to the involvement of ET signalling in AtPDF1.1-mediated tolerance to *Pcc* infection.

## Discussion

Iron is an essential nutrient for hosts and pathogens^21^ and so the iron status of a plant can affect the pathogenesis of pathogens^31^. One of the first lines of defence against bacterial infection is to sequester and withhold iron in an iron-storage compartment, limiting bacterial growth and pathogenicity^49^. In this study, we found that transcription levels of *AtPDF1*.*1* are elevated during *Pcc* infection (Fig. S2) and that the AtPDF1.1 protein is secreted into the apoplast to sequester iron (Figs 2 and 3 and S11). We propose that this process serves as a key defence mechanism, limiting iron availability and thereby compromising the pathogenesis of *Pcc*. This hypothesis was supported by the increased sensitivity to *Pcc* invasion of the *AtPDF1*.*1* OE plants after iron supplementation (Fig. S5).

The state of iron deficiency in *AtPDF1*.*1* OE plants leads to the induction of ET signalling in the roots (Fig. 6)^46, 47^, which can then activate the ERF branch, but not the MYC branch, of the JA/ET-mediated response in systemic leaves (Fig. 5). Since the JA/ET signalling pathway is involved in the defence against *Pcc* ^44, 45^, the ability of AtPDF1.1 to systemically induce an iron-deficiency-mediated ET response would be one of its functions in defending off *Pcc*. The fact that blocking ET signalling led to increased susceptibility to *Pcc* infection (Fig. S15) further reinforces the involvement of ET signalling in AtPDF1.1-mediated tolerance to *Pcc* infection.

Our study showed that AtPDF1.1 could chelate iron (Figs 3 and S11) and its overexpression caused an iron deficiency response (Fig. 4), which correlates well with the promoted tolerance to *Pcc* infection (Figs 1 and S5). These results mirror previous studies which demonstrated that iron deficiency triggers defence responses and prompts resistance against pathogenic microbes. For instance, Koen *et al*.^50^ showed that treatment of β-aminobutyric acid (BABA), a nonprotein amino acid able to chelate iron, led to a transient iron deficiency response which is linked with a protective effect against microbial pathogens. In addition, siderophore pyoverdine, which is produced by the beneficial bacterium *Pseudomonas fluorescens*, restored the growth of *A*. *thaliana* under iron-deficient conditions by repressing defence pathways and activating iron acquisition/redistribution signalling^51^, further demonstrating the link between iron deficiency and immune responses.

During *Pcc* infection, genes associated with both JA/ET- and SA-mediated defence responses are activated^52^. In this study, we showed that plants ectopically expressing *AtPDF1*.*1* had pre-existing protection against *Pcc* infection (Fig. 1) and exhibited an iron deficiency state (Figs 4 and S8). In addition, the iron-deficiency-mediated JA/ET response is further enhanced upon *Pcc* infection (Fig. S12A), suggesting that AtPDF1.1 has a synergistic effect on the *Pcc*-mediated iron deficiency response, and is capable of amplifying the ET defence response. *Pcc* infection also led to decreased iron content in root apoplasts (Fig. S7A) and activated JA/ET signalling (Fig. S12A). Therefore, exposing plants to AtPDF1.1 prior to pathogen infection may have a protective and amplifying effect on their disease defence responses.

SA-mediated defence responses are involved in preventing infection by the hemibiotrophic bacterial pathogen *Pst* DC3000^53^. Bacterial siderophores induce an iron-deficiency-mediated SA immune response to defend against *Pst* DC3000 in *A*. *thaliana* by restricting Fe^3+^ availability in the leaf cell wall. This restriction results in a perturbation of iron distribution and activates an iron deficiency response^23^. Although AtPDF1.1 also has an iron-sequestration action, and is capable of inducing an iron-deficiency-mediated response. The expression of *AtPDF1*.*1* does not affect defence against *Pst* DC3000 infection (Fig. S13) nor the activation of genes involved in SA signalling (Fig. 5B). Interestingly, siderophore pyoverdine produced by the beneficial bacterium *Pseudomonas fluorescens* has an opposite effect, in which pyoverdine promoted *Arabidopsis* growth under iron-deficient conditions by repressing defence pathways, including SA and abscisic acid, leading to increased susceptibility to *Botrytis cinerea* ^52^. Taken together, these results indicate that the iron-deficiency-mediated signalling triggered by AtPDF1.1 specifically enhances the ET-mediated defence response. The differences in the immune response pathways activated by the AtPDF1.1 protein and siderophores may be due to their different properties. First, the cysteine-rich AtPDF1.1 utilizes sulphur (S) atoms as an electron donor to bind Fe^2+^ and Fe^3+^ ions (Fig. S6B), whereas siderophores predominantly chelate Fe^3+^ via their oxygen (O) ligands^22, 23, 54, 55^. In addition, *AtPDF1*.*1* is induced systemically during *Pcc* infection (Fig. S2B), whereas siderophores are not proteins and are secreted by pathogens at the infection site^22^.

The fact that *AtPDF1*.*1* overexpression resulted in high levels of apoplastic iron accumulation (Fig. 3), which was accompanied by a decrease in intracellular iron content, and reduced *AtPDF1*.*1* expression gave an opposite effect (Fig. S7) suggests that the chelation of iron by AtPDF1.1 in the apoplast influences intracellular iron levels. It has been reported that coumarin phenolic compounds and nicotianamine can chelate apoplastic iron ions and are required for iron uptake from roots^41, 56, 57^. We postulate that high levels of AtPDF1.1 proteins present in the apoplast may compete for iron ions with coumarin phenolic compounds and nicotianamine, resulting in the sequestration of iron in the apoplast (Figs 3 and S11). In addition, siderophore-mediated-upregulation of *AtFer1* has been identified as an iron-withholding defence against the proliferation of the necrotrophic bacterium *D*. *dadantii* ^33^. We propose that AtPDF1.1 may similarly compete with bacterial siderophores for iron binding and thereby mediate an iron-withholding defence against *Pcc* invasion.

**Figure 7:**
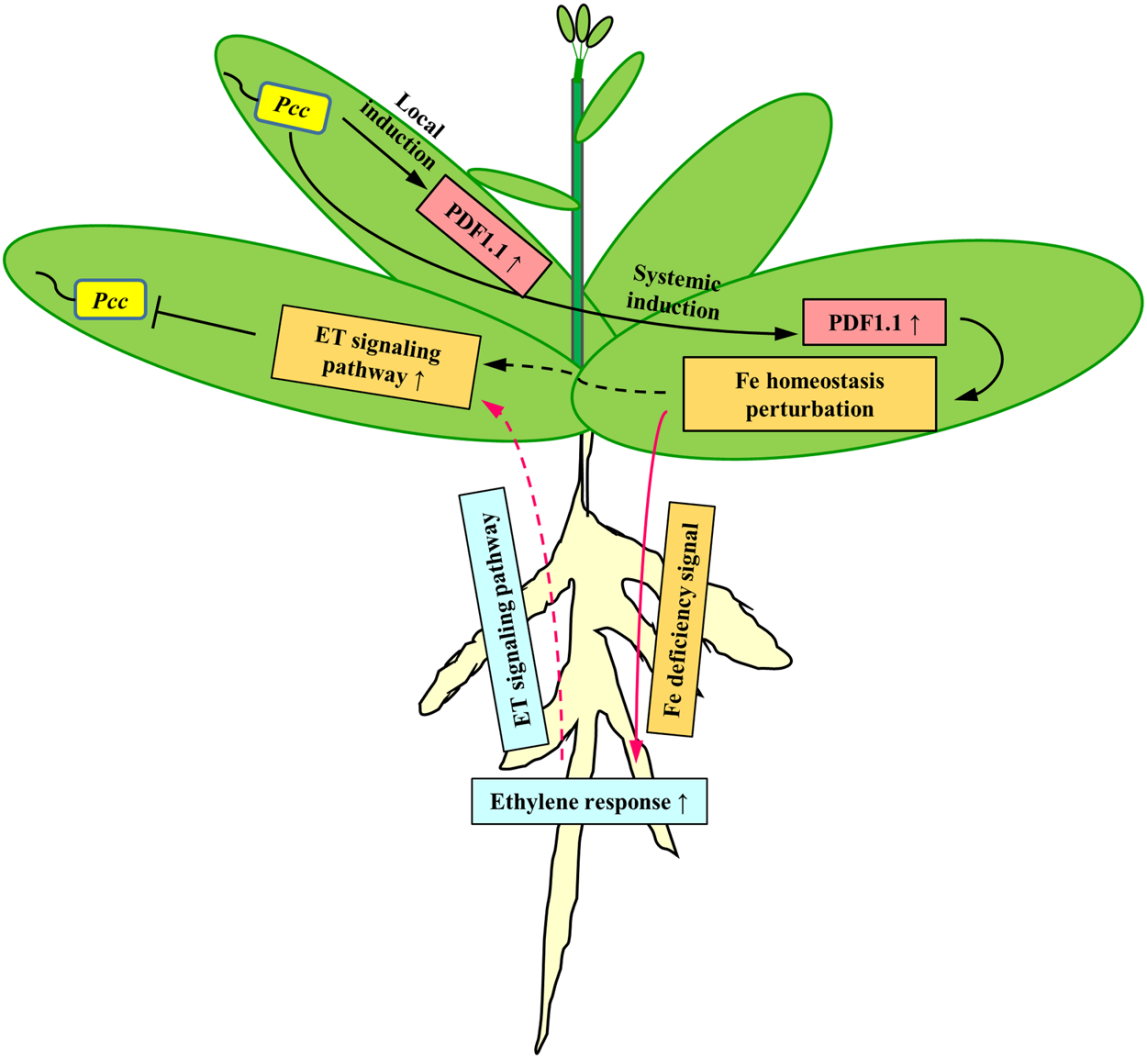
A proposed model of *AtPDF1*.*1*-mediated defence response to the infection by *Pcc*. During *Pcc* infection, transcription of *AtPDF1*.*1* is upregulated in both the local infected and systemic non-infected leaves. The production of AtPDF1.1 protein leads to the chelation/restriction of iron availability in the apoplast, and the consequent perturbation of iron homeostasis. This results in the activation of an iron deficiency response in leaves and the subsequent activation of the ethylene (ET) biosynthesis and signalling pathway in roots. This in turn causes the systemic activation of a jasmonic acid (JA)/ET-mediated immune response in leaves, which promotes tolerance to the infection of necrotrophic pathogens.

In addition to its known antifungal function^58^, this study shows that AtPDF1.1 possesses an iron-withholding defence function to limit the growth of the necrotrophic bacterial pathogen *Pcc* via the activation of the ET defence pathway (Fig. 7). During *Pcc* infection, signals induced by the pathogen may trigger the translocation of iron ions from the root apoplast to the leaf apoplast for bacterial utilization and proliferation. As a defence strategy, plants elevate *AtPDF1*.*1* expression, and apoplast-localized AtPDF1.1 chelates iron, thereby iron homeostasis is perturbed to activate an iron deficiency response in the leaf. In addition, this may potentially lead to the activation of ET signalling and biosynthesis in the roots, and subsequently trigger an ET-mediated defence response in systemic leaves to counteract the further attack of pathogens^45, 59^. The pre-existing ET defence response mediated by *AtPDF1*.*1* in the *AtPDF1*.*1* OE plants may account for the increased tolerance to *Pcc* infection, while suppressing *AtPDF1*.*1* expression reduces defence against *Pcc* infection.

## Methods

### Plant materials and growth conditions

The growth conditions and selection of *Arabidopsis* are described in Method S1.

### Generation of transgenic plants

The constructions of AtPDF1.1 OE (OE), AtPDF1.1ΔSP (ΔSP), AtPDF1.1 RNAi, AtPDF1.1:GFP, AtPDF1.1ΔSP:GFP, AtPDF1.1 (SP only):GFP and Arabidopsis transformation are described in Method S2.

### Gene expression analysis following metal treatments and *Pcc* infection

Ten-day old seedlings of Ev plants were cultured on ½ MS medium supplemented with 600 μM ZnSO_4_, 600 μM FeSO_4_, 75 μM CuSO_4_ or 300 μM ferrozine (Aldrich,160601) for 0 to 5 days. Four-week old soil-grown Ev plants were punctured with a 5 ml syringe needle and inoculated with or without 3 μl liquid culture of 5 × 10^6^ cfu ml^−1^ of *Pcc* (three rosette leaves/plant) and local and systemic leaves were harvested at 1–5 hours or 4–24 hours post inoculation (hpi). Total RNA was isolated using the Trizol RNA isolation reagent (Invitrogen), according to the manufacturer’s instructions. cDNAs were synthesized using the M-MLV reverse transcriptase cDNA synthesis kit (Promega) according to the supplier’s instructions. *Actin2* (AT3G18780) or *UBC21* (AT5G25760) were used as internal normalization controls. qPCR was performed as described by Ko *et al*.^60^. The primers used to analyse *AhPDF1s* have already been described by Shahzad *et al*.^11^. Primers used for gene expression studies are listed in Table S2.

### Plant pathogen inoculation and disease response assay

For the *Pcc* infection assay, bacteria were grown at 28 °C overnight in Luria-Bertani (LB) medium (Difco, 244620). Three rosette leaves from each four-week old soil-grown plant were punctured with a 5 ml syringe needle and inoculated with 3 μl liquid culture of 5 × 10^6^ cfu ml^−1^ of *Pcc* or water (control). Bacterial soft rot symptoms were evaluated periodically. The maximal lesion sizes (diameter; mm) on leaves were recorded at 24 hpi using the protocol described by Li *et al*.^61^. For the *P*. *syringae* pv. *tomato* (*Pst*) DC3000 infection assay, the bacteria were grown at 28 °C overnight in King’s B medium containing 50 μg ml^−1^ rifampicin^62^. Plants were sprayed with liquid culture of 2 × 10^8^ cfu mL^−1^ of *Pst* DC3000 containing 0.02% Silwet L-77 or 10 mM MgSO_4_ (control). Disease symptoms were evaluated periodically and *in planta* bacterial CFU counts in leaves were measured at 3 dpi.

### Subcellular protein localization

Ten-day-old transgenic plants were immersed in 1× phosphate-buffered saline (pH 7.4; MDBio) for 30 min to induce plasmolysis and to stabilize EGFP fusion proteins in the apoplast^63, 64^. The true leaves were removed and imaging was performed using a Zeiss confocal microscope LSM 710 (Zeiss Axio Observer Z.1) with a Plan-Apochromat 100×/1.40 Oil DIC M27 objective lens. For detection of GFP signals, a 488 nm excitation wavelength and a 500–550 nm emission wavelength were used.

### Detection of secreted proteins

Suspension cells of transgenic plants were induced as described by May and Leaver^65^. The culture media were harvested weekly and combined. Each 200 ml of culture media was centrifuged at 8,000 *g* for 10 min to remove debris, and filtered through Miracloth (Calbiochem) and two layers of Whatman grade 1 qualitative filter paper. The filtrates were lyophilized using a freeze dryer (Uniss), and dissolved in 10 ml milli-Q H_2_O and then desalted and concentrated to 10-fold volume using Vivaspin 6 centrifugal filters (Sartorius). The protein concentrations in the different samples were measured by protein assay dye reagent (Bio-Rad [5000006]) and adjusted to the same value with SDS sample buffer^66^ and 20 µg protein per sample was used for immunoblot analysis.

### Immunoblot analysis

Experimental procedures are described in Method S3.

### Metal binding assay

The experimental design and procedure are described in Method S4.

### Detection of iron distribution

For Perls staining, roots were vacuum infiltrated with Perls staining solution containing equal volumes of 4% (v/v) HCl and 4% (w/v) K-ferrocyanide for 15 min and incubated at room temperature for 30 min^67^. The formation of blue pigments, denoting iron accumulation, was observed using light microscopy. For Perls/DAB/H_2_O_2_ staining, leaves and roots were vacuum infiltrated with fixation solution and dehydrated as described by Roschzttardtz *et al*.^68^. Embedding and sectioning were performed as described by Lin *et al*.^69^. Sections for infected leaves (7 mm from the inoculation site), systemic leaves and roots were subjected to Perls/DAB/H_2_O_2_ staining^68^ and imaging using a Zeiss LSM 710 confocal microscope fitted with an EC Plan-Neofluar 20×/0.50 M27 lens, under transmitted light (bright field).

### Measurement of metal concentration

The experimental design and procedure are described in Method S5.

### FCR activity assays

FCR activity was measured in four-week-old transgenic plants grown in hydroponic medium. Three leaves from each plant were punctured and inoculated with 3 μl liquid culture of 5 × 10^6^ cfu ml^−1^ of *Pcc* or water (control). The roots were harvested at 24 hpi and a modified FCR activity assay was performed as previously described^70^.

### Iron infiltration and treatment of ethylene inhibitor

The 2.5 mM MES buffer, pH 5.6 containing with or without FeSO_4_ was infiltrated into the *Arabidopsis* leaves using 1 ml syringes without a needle, and followed by inoculation of *Pcc* 1 hour after iron infiltration.

Four-week-old soil-grown transgenic plants were treated with ethylene inhibitor, 1-methylcyclopropene (1-MCP, Lytone Enterprise, one tablet containing 2 mg 1-MCP was dissolved in 15 ml milli-Q H_2_O) in a 150 L sealed chamber for 24 hours, followed by inoculation with *Pcc*.

### Accession numbers

The sequences of genes used in this article were obtained from The *Arabidopsis* Information Resource (TAIR, http://www.arabidopsis.org/index.jsp). The accession numbers of the genes are described in Method S6.

## Electronic supplementary material

supplementary information

## References

[1] Pieterse CMJ, Leon-Reyes A, Van der Ent S, Van Wees SCM (2009). Networking by small-molecule hormones in plant immunity. Nat. Chem. Biol..

[2] Raub JA, Mathieu-Nolf M, Hampson NB, Thom SR (2000). Carbon monoxide poisoning–a public health perspective. Toxicology.

[3] Janeway CA (1989). Approaching the asymptote? Evolution and revolution in immunology. Cold Spring Harb. Symp. Quant. Biol..

[4] De Coninck B, Cammue BPA, Thevissen K (2013). Modes of antifungal action and in planta functions of plant defensins and defensin-like peptides. Fungal Biol. Rev..

[5] Dias RO, Franco OL (2015). Cysteine-stabilized alphabeta defensins: From a common fold to antibacterial activity. Peptides.

[6] Marmiroli N, Maestri E (2014). Plant peptides in defence and signalling. Peptides.

[7] Tam J, Wang S, Wong K, Tan W (2015). Antimicrobial peptides from plants. Pharmaceuticals.

[8] Vriens K, Cammue BP, Thevissen K (2014). Antifungal plant defensins: mechanisms of action and production. Molecules.

[9] Kaur J, Sagaram US, Shah D (2011). Can plant defensins be used to engineer durable commercially useful fungal resistance in crop plants?. Fungal Biol. Rev..

[10] Mirouze M (2006). A putative novel role for plant defensins: a defensin from the zinc hyper-accumulating plant, *Arabidopsis halleri*, confers zinc tolerance. Plant J..

[11] Shahzad Z (2013). *Plant Defensin type 1* (*PDF1*): protein promiscuity and expression variation within the *Arabidopsis* genus shed light on zinc tolerance acquisition in *Arabidopsis halleri*. New Phytol..

[12] Silverstein KA, Graham MA, Paape TD, VandenBosch KA (2005). Genome organization of more than 300 defensin-like genes in *Arabidopsis*. Plant Physiol..

[13] Terras FR (1993). A new family of basic cysteine-rich plant antifungal proteins from Brassicaceae species. FEBS Lett..

[14] Zimmerli L, Stein M, Lipka V, Schulze-Lefert P, Somerville S (2004). Host and non-host pathogens elicit different jasmonate/ethylene responses in *Arabidopsis*. Plant J..

[15] Nguyen NN (2014). Evolutionary tinkering of the expression of PDF1s suggests their joint effect on zinc tolerance and the response to pathogen attack. Front. Plant Sci..

[16] Kobayashi T, Nishizawa NK (2012). Iron uptake, translocation, and regulation in higher plants. Annu. Rev. Plant Biol..

[17] Briat JF, Curie C, Gaymard F (2007). Iron utilization and metabolism in plants. Curr. Opin. Plant Biol..

[18] Shin LJ (2013). IRT1 degradation factor1, a ring E3 ubiquitin ligase, regulates the degradation of iron-regulated transporter1 in *Arabidopsis*. Plant Cell.

[19] Connolly EL, Fett JP, Guerinot ML (2002). Expression of the *IRT1* metal transporter is controlled by metals at the levels of transcript and protein accumulation. Plant Cell.

[20] Yuan Y (2008). FIT interacts with AtbHLH38 and AtbHLH39 in regulating iron uptake gene expression for iron homeostasis in *Arabidopsis*. Cell Res..

[21] Costa A (2015). AIR12, a b-type cytochrome of the plasma membrane of *Arabidopsis thaliana* is a negative regulator of resistance against *Botrytis cinerea*. Plant Sci..

[22] Winkelmann G (2007). Ecology of siderophores with special reference to the fungi. Biometals.

[23] Aznar A (2014). Scavenging iron: a novel mechanism of plant immunity activation by microbial siderophores. Plant Physiol..

[24] Kim HS (2014). Overexpression of the *Brassica rapa* transcription factor WRKY12 results in reduced soft rot symptoms caused by *Pectobacterium carotovorum* in *Arabidopsis* and *Chinese cabbage*. Plant Biol. (Stuttg.).

[25] Bendtsen JD, Nielsen H, von Heijne G, Brunak S (2004). Improved prediction of signal peptides: SignalP 3.0. J. Mol. Biol..

[26] Oomen RJ (2011). Plant defensin AhPDF1.1 is not secreted in leaves but it accumulates in intracellular compartments. New Phytol..

[27] Terras FR (1995). Small cysteine-rich antifungal proteins from radish: their role in host defence. Plant Cell.

[28] Amien S (2010). Defensin-like ZmES4 mediates pollen tube burst in maize via opening of the potassium channel KZM1. PLoS Biol..

[29] Toth IK (1999). Evaluation of phenotypic and molecular typing techniques for determining diversity in *Erwinia carotovora* subspp. *atroseptica*. J. Appl. Microbiol..

[30] Yu X (2014). Transcriptional analysis of the global regulatory networks active in *Pseudomonas syringae* during leaf colonization. MBio.

[31] Kieu NP (2012). Iron deficiency affects plant defence responses and confers resistance to *Dickeya dadantii* and *Botrytis cinerea*. Mol. Plant Pathol..

[32] Franza T, Mahe B, Expert D (2005). *Erwinia chrysanthemi* requires a second iron transport route dependent of the siderophore achromobactin for extracellular growth and plant infection. Mol. Microbiol..

[33] Dellagi A (2005). Siderophore-mediated upregulation of *Arabidopsis* ferritin expression in response to *Erwinia chrysanthemi* infection. Plant J..

[34] Oshima T, Oshima C, Baba Y (2015). Selective extraction of histidine derivatives by metal affinity with a copper (II)-chelating ligand complex in an aqueous two-phase system. J. Chromatogr. B Analyt. Technol. Biomed. Life Sci..

[35] Kavaklı PA, Kavaklı C, Güven O (2014). Preparation and characterization of Fe (III)-loaded iminodiacetic acid modified GMA grafted nonwoven fabric adsorbent for anion adsorption. Radiat. Phys. Chem..

[36] Das A (1990). Stabilities of ternary complexes of cobalt (II), nickel (II), copper (II) and zinc (II) involving aminopolycarboxylic acids and heteroaromatic N-bases as primary ligands and benzohydroxamic acid as a secondary ligand. Transit. Metal. Chem..

[37] Lei GJ (2014). Abscisic acid alleviates iron deficiency by promoting root iron reutilization and transport from root to shoot in *Arabidopsis*. Plant Cell Environ..

[38] Bienfait HF, van den Briel W, Mesland-Mul NT (1985). Free space iron pools in roots: generation and mobilization. Plant Physiol..

[39] Pushnik JC, Miller GW, Manwaring JH (1984). The role of iron in higher plant chlorophyll biosynthesis, maintenance and chloroplast biogenesis. J. Plant Nutr..

[40] Sivitz AB, Hermand V, Curie C, Vert G (2012). *Arabidopsis* bHLH100 and bHLH101 control iron homeostasis via a FIT-independent pathway. PLoS One.

[41] Koen E (2013). *Arabidopsis thaliana* nicotianamine synthase 4 is required for proper response to iron deficiency and to cadmium exposure. Plant Sci..

[42] Wu H (2012). Co-overexpression *FIT* with *AtbHLH38* or *AtbHLH39* in *Arabidopsis*-enhanced cadmium tolerance via increased cadmium sequestration in roots and improved iron homeostasis of shoots. Plant Physiol..

[43] Robinson NJ, Procter CM, Connolly EL, Guerinot ML (1999). A ferric-chelate reductase for iron uptake from soils. Nature.

[44] Anderson JP (2004). Antagonistic interaction between abscisic acid and jasmonate-ethylene signalling pathways modulates defence gene expression and disease resistance in *Arabidopsis*. Plant Cell.

[45] Pre M (2008). The AP2/ERF domain transcription factor ORA59 integrates jasmonic acid and ethylene signals in plant defence. Plant Physiol..

[46] Garcia MJ, Suarez V, Romera FJ, Alcantara E, Perez-Vicente R (2011). A new model involving ethylene, nitric oxide and Fe to explain the regulation of Fe-acquisition genes in Strategy I plants. Plant Physiol. Biochem..

[47] Garcia MJ, Lucena C, Romera FJ, Alcantara E, Perez-Vicente R (2010). Ethylene and nitric oxide involvement in the up-regulation of key genes related to iron acquisition and homeostasis in *Arabidopsis*. J. Exp. Bot..

[48] Walley JW (2008). The chromatin remodeler SPLAYED regulates specific stress signalling pathways. PLoS Pathog..

[49] Fones H, Preston GM (2013). The impact of transition metals on bacterial plant disease. FEMS Microbiol. Rev..

[50] Trapet P (2016). The *Pseudomonas fluorescens* siderophore pyoverdine weakens *Arabidopsis thaliana* defense in favor of growth in iron-deficient conditions. Plant Physiol..

[51] Koen E (2014). β-Aminobutyric acid (BABA)-induced resistance in *Arabidopsis thaliana*: link with iron homeostasis. Mol. Plant Microbe Interact..

[52] Po-Wen C, Singh P, Zimmerli L (2013). Priming of the *Arabidopsis* pattern-triggered immunity response upon infection by necrotrophic *Pectobacterium carotovorum* bacteria. Mol. Plant Pathol..

[53] Nawrath C, Metraux JP (1999). Salicylic acid induction-deficient mutants of *Arabidopsis* express *PR*-*2* and *PR*-5 and accumulate high levels of camalexin after pathogen inoculation. Plant Cell.

[54] Miethke M, Marahiel MA (2007). Siderophore-based iron acquisition and pathogen control. Microbiol. Mol. Biol. Rev..

[55] Alvarez-Fernandez A, Diaz-Benito P, Abadia A, Lopez-Millan AF, Abadia J (2014). Metal species involved in long distance metal transport in plants. Front. Plant. Sci..

[56] Fourcroy P (2014). Involvement of the ABCG37 transporter in secretion of scopoletin and derivatives by *Arabidopsis* roots in response to iron deficiency. New Phytol..

[57] Fourcroy P, Tissot N, Gaymard F, Briat JF, Dubos C (2016). Facilitated Fe nutrition by phenolic compounds excreted by the *Arabidopsis* ABCG37/PDR9 transporter requires the IRT1/FRO2 high-affinity root Fe^2+^ transport system. Mol. Plant.

[58] De Coninck BM (2010). *Arabidopsis thaliana* plant defensin AtPDF1.1 is involved in the plant response to biotic stress. New Phytol..

[59] Glazebrook J (2005). Contrasting mechanisms of defence against biotrophic and necrotrophic pathogens. Annu. Rev. Phytopathol..

[60] Ko SS (2014). The bHLH142 transcription factor coordinates with TDR1 to modulate the expression of *EAT1* and regulate pollen development in rice. Plant Cell.

[61] Li CW (2011). Tomato RAV transcription factor is a pivotal modulator involved in the AP2/EREBP-mediated defence pathway. Plant Physiol..

[62] King EO, Ward MK, Raney DE (1954). Two simple media for the demonstration of pyocyanin and fluorescin. J. Lab. Clin. Med..

[63] Yu, Q., Tang, C. & Kuo, J. A critical review on methods to measure apoplastic pH in plants. *Plant Soil* **219**, 29–40 (2000).

[64] Patterson GH, Knobel SM, Sharif WD, Kain SR, Piston DW (1997). Use of the green fluorescent protein and its mutants in quantitative fluorescence microscopy. Biophys. J..

[65] May MJ, Leaver CJ (1993). Oxidative stimulation of glutathione synthesis in *Arabidopsis thaliana* suspension cultures. Plant Physiol..

[66] Laemmli UK (1970). Cleavage of structural proteins during the assembly of the head of bacteriophage T4. Nature.

[67] Soeters R, Aus C (1989). Hazards of injectable therapy. Trop. Doct..

[68] Roschzttardtz H (2013). New insights into Fe localization in plant tissues. Front. Plant Sci..

[69] Lin HY (2014). Genome-wide annotation, expression profiling, and protein interaction studies of the core cell-cycle genes in *Phalaenopsis aphrodite*. Plant Mol. Biol..

[70] Yi Y, Guerinot ML (1996). Genetic evidence that induction of root Fe (III) chelate reductase activity is necessary for iron uptake under iron deficiency. Plant J..

