# Supplementary material for: The Arabidopsis defensin gene, AtPDF1.1, mediates defence against Pectobacterium carotovorum subsp. carotovorum via an iron-withholding defence system
Source: Sci Rep. 2017 Aug 23;7:9175. doi: 10.1038/s41598-017-08497-7 (PMC5569111; doi:10.1038/s41598-017-08497-7)
Supplement: Supplementary file 1 — supplementary information [file 41598_2017_8497_MOESM1_ESM.pdf]

Scientific Reports Supplementary Information

The *Arabidopsis* defensin gene, *AtPDF1.1*, mediates defence against *Pectobacterium carotovorum* subsp. *carotovorum* via an iron-withholding defence system

Pao-Yuan Hsiao, Chiu-Ping Cheng*, Kah Wee Koh, Ming-Tsair Chan*

The following additional information is available for this article:

Figure S1. Expression levels of *AtPDF1s* are increased by treatment with zinc, iron or copper.

Figure S2. Expression levels of *AtPDF1.1* were increased both locally and systemically by *Pcc* infection.

Figure S3. Prediction plot of the AtPDF1.1 signal peptide and expression of *AtPDF1.1* in transgenic plants.

Figure S4. AtPDF1.1 protein is a secreted protein identified in the culture medium.

Figure S5. Infiltration of iron significantly increases *Pcc*-mediated symptoms in all transgenic plants.

Figure S6. AtPDF1.1 protein potentially binds to iron ions.

Figure S7. AtPDF1.1 protein tends to accumulate irons in the apoplast.

Figure S8. *AtPDF1.1* OE plants had less chlorophyll content than the other transgenic plants.

Figure S9. Iron deficiency-associated genes are increased after infection of *Pcc*.

Figure S10. Ferric chelate reductase (FCR) activities are enhanced with expression of *AtPDF1.1* and infection of *Pcc*.

Figure S11. *Pcc* infection induced apoplastic iron accumulation both locally and systemically.

Figure S12. Downstream genes of *ERF1*/*2* are activated by *Pcc* infection .

Figure S13. *AtPDF1.1* transgenic plants do not confer protection against *P. syringae* pv. *tomato* DC3000.

Figure S14. ET -biosynthesis and signalling genes are up-regulated by infection of *Pcc*.

Figure S15. Application of ethylene inhibitor significantly enhances the severity of *Pcc*-mediated disease.

Table S1. List of primers used for vector construction.

Table S2. List of primers used to quantify gene expression levels.

Method S1. Plant materials and growth conditions.

Method S2. Generation of transgenic plants.

Method S3. Immunoblot analysis.

Method S4. Metal binding assay.

Method S5. Measurement of metal concentration.

Method S6. Accession numbers.

Supplementary references

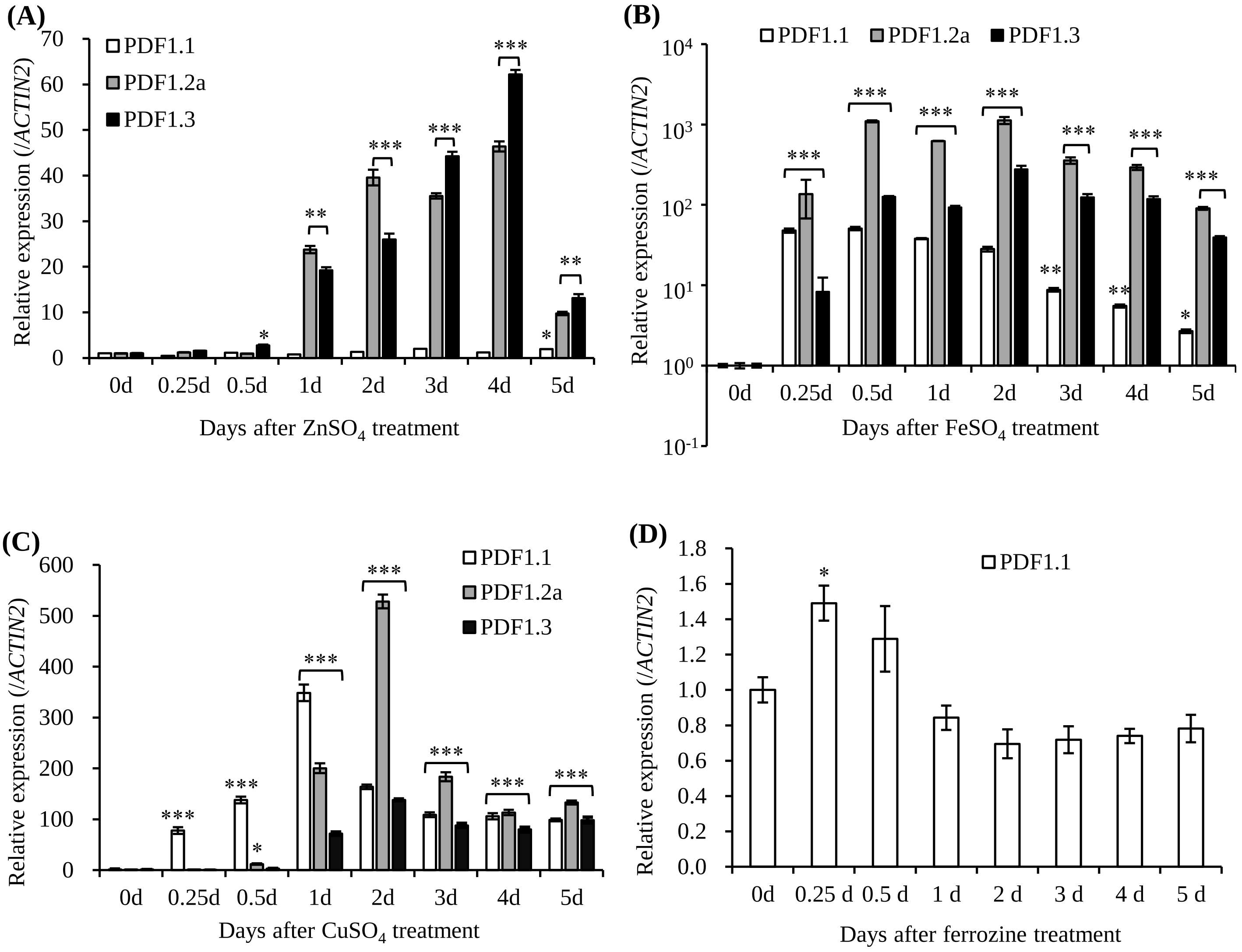

Figure S1. Expression levels of *AtPDF1s* are increased by treatment with zinc, iron or copper. Seedlings of pH2GW7 empty-vector control transgenic *Arabidopsis thaliana* plants (Ev) were treated with 600 μM $ZnSO_4$ (**A**), 600 μM $FeSO_4$ (**B**), 75 μM $CuSO_4$ (**C**) or 300 μM ferrozine (**D**) for 0 to 5 days. The relative expression levels of *AtPDF1s* were monitored by qPCR using cDNA derived from whole plants. Transcript levels were normalized to those of the internal control gene *ACTIN2* and compared with the non-treated control (0 d, defined as a value of 1). Values are means ± standard errors from fifteen seedlings for each treatment and each time point was assayed in a single experiment that was repeated at least three times with similar results. Asterisks (*) above each bar indicate significant difference (Fisher's Least Significant Difference (LSD) post hoc one-way ANOVA, * $P < 0.05$, ** $P < 0.01$, *** $P < 0.001$) relative to 0 d.

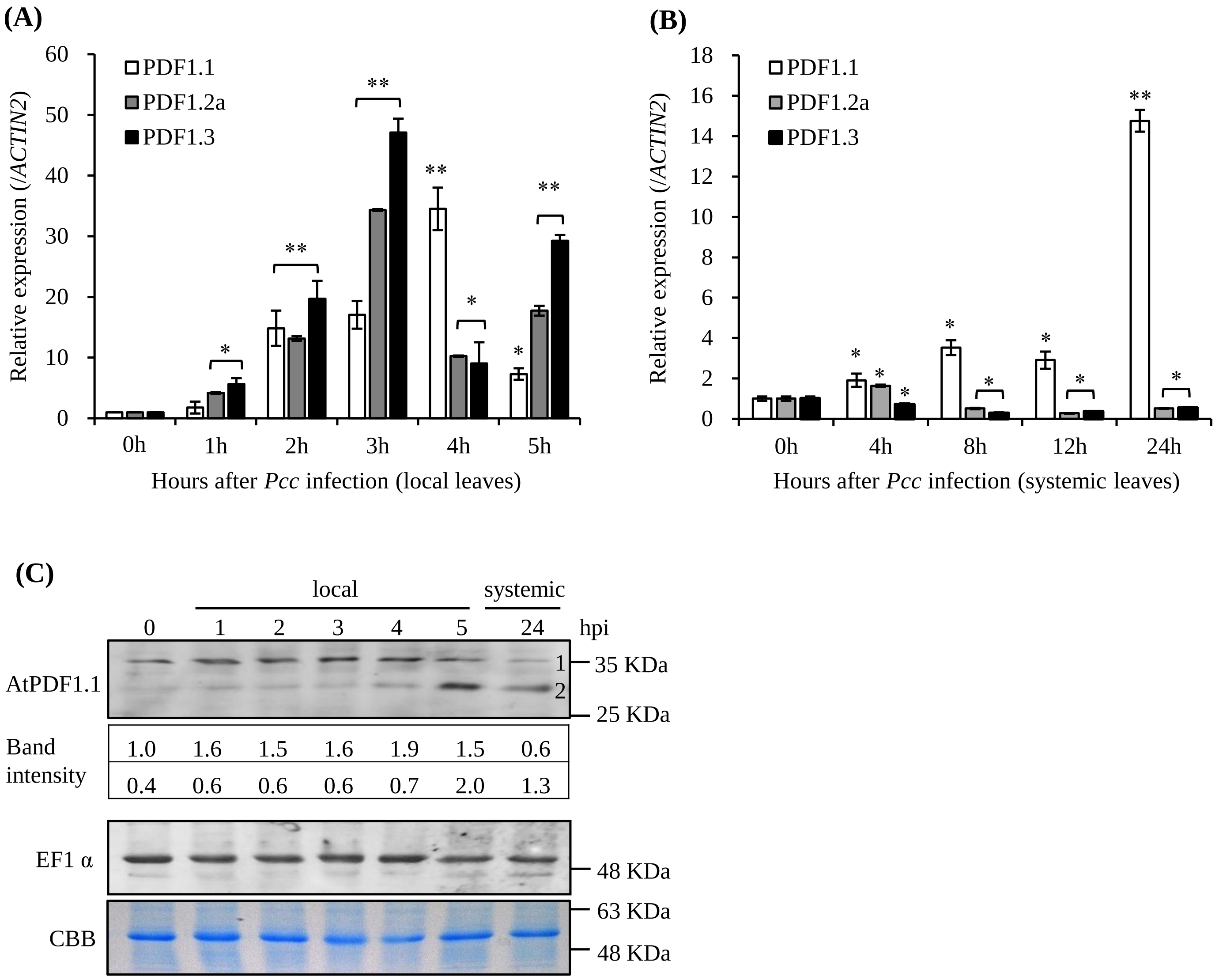

Figure S2. Expression levels of *AtPDF1.1* were increased both locally and systemically by *Pcc* infection. Leaves of pH2GW7 empty-vector plants (Ev) were inoculated with *Pcc* and harvested at different time points. The relative expression levels of *AtPDF1.1*, *AtPDF1.2a* and *AtPDF1.3* in the local (**A**) or systemic leaves (**B**) were monitored by qPCR, and compared with a water-treated control (0 h, defined value of 1). The transcript levels were normalized to those of *ACTIN2*. Values are means ± standard errors from eight samples for each infection and each time point in a single experiment that was repeated at least three times with similar results. Asterisk (*) above each bar indicate significant difference (LSD post hoc one-way ANOVA, * $P < 0.05$, ** $P < 0.01$) relative to 0 h. (**C**) The pro*AtPDF1.1::AtPDF1.1:GFP* (green fluorescent protein) transgenic *Arabidopsis thaliana* plants were inoculated with *Pcc*, and the local and systemic leaves were harvested at 1-5 hpi or 24 hpi, respectively. Total protein extracts were subjected to immunoblot analysis with anti-AtPDF1.1 and anti-EF1α antibodies (control for expression). One (1) denotes the putative full-length AtPDF1.1: GFP protein and 2 denotes the putative AtPDF1.1: GFP protein with the N-terminal signal peptide being cleaved. The relative band intensity of each band was calculated by ImageJ. Each lane was loaded with 20 µg of protein, and equal loading was validated using Coomassie blue staining (bottom panel).

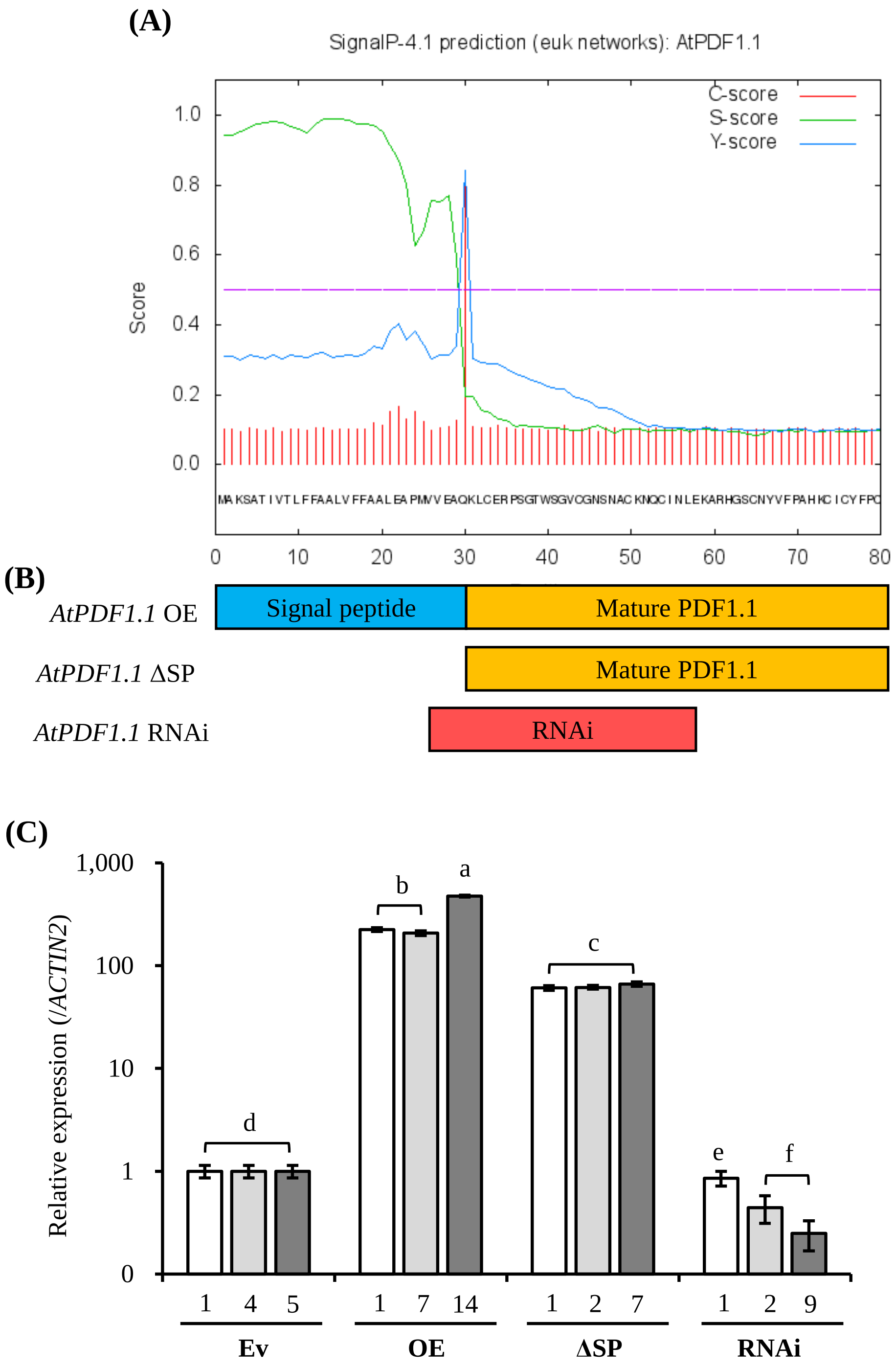

Figure S3. Prediction plot of the AtPDF1.1 signal peptide and expression of *AtPDF1.1* in transgenic plants. (**A**) The amino acid sequence of the *AtPDF1.1*-encoding gene was analysed using SignalP-4.1 (http://www.cbs.dtu.dk/services/SignalP). The graph shows the cleavage site score (C-score, cleavage site score denoting a putative cleavage site in the sequence) and the signal peptide score (S-score, signal peptide score denoting the probability that an amino acid is part of a signal peptide). The Y-score denotes the combination of the C-score and S-score and the predicted N-terminal signal peptide with a cleavage site within position 29 and 30 (C score, red plot; S score, green plot; Y score, blue plot). (**B**) A schematic diagram of the AtPDF1.1 protein with the position of the signal peptide (1-29 amino acids, blue bar) and mature AtPDF1.1 peptide (30-80 amino acids, yellow bar) are indicated. The nucleotides at position 77-173 (red bar) from the start codon were used to generate the RNAi construct. (**C**) The relative expression levels of *PDF1.1* were measured in three independent ten-day-old T_3 seedlings of Ev (empty vector), *AtPDF1.1* OE (overexpression), *AtPDF1.1*ΔSP OE (ΔSP) and *AtPDF1.1* RNAi (RNAi) transgenic plants by qPCR, and compared with expression in empty vector control plants (Ev, defined as a value of 1). Transcription levels were normalized to those of *ACTIN2*. Values are means ± standard errors from fifteen seedlings for each line in a single experiment that was repeated at least three times with similar results. Different letters above each bar indicate significant difference (LSD post hoc one-way ANOVA, $P < 0.05$).

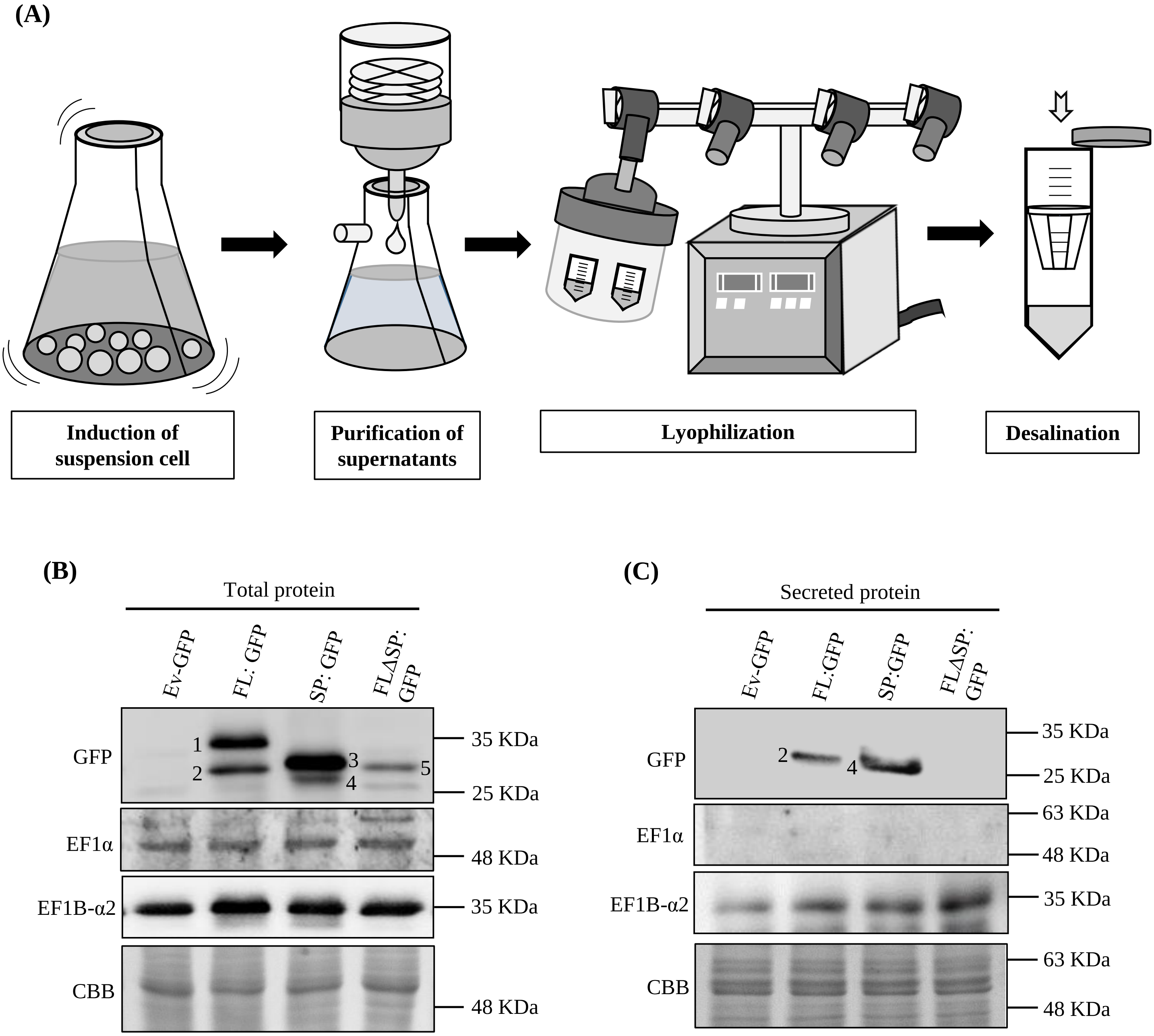

Figure S4. AtPDF1.1 protein is a secreted protein identified in the culture medium. (**A**) A schematic diagram illustrating the procedure used to purify and concentrate the secreted proteins from the suspension cell system[65]. The purified proteins were adjusted to the same concentration with SDS sample buffer for immunoblot analysis. Total proteins (**B**) and secreted proteins (**C**) harvested from transgenic *A. thaliana* plants overexpressing pH7FWG2 empty vector (Ev-GFP), *AtPDF1.1:GFP* (FL:GFP), *AtPDF1.1(SP only):GFP* (SP:GFP) and *AtPDF1.1ΔSP:GFP* (FLΔSP:GFP) (described in Fig. 4A) were analysed by immunoblot analysis. Each line was repeated at least three times with similar results. One (1) denotes AtPDF1.1:GFP protein; 3 denotes AtPDF1.1(SP only):GFP protein; 5 denotes AtPDF1.1ΔSP: GFP protein; and 2 and 4 denote that signal peptides were cleaved in the AtPDF1.1: GFP and AtPDF1.1(SP only):GFP protein, respectively. The GFP fusion protein (top panel), elongation factor 1α (EF1α; 2nd panel) and EF1B-α2 (3rd panel) were visualized using anti-GFP, anti-EF1α (control for expression) or anti-EF1B-α2 (control for expression) antibodies, respectively. A total of 20 μg of protein was loaded and validated by Coomassie blue staining (bottom panel). The expected size of each protein: AtPDF1.1:GFP (35.5 KDa, band 1), AtPDF1.1ΔSP:GFP (32 KDa, band 2 and 5), AtPDF1.1(SP only):GFP (30.5 KDa, band 3), GFP (27 KDa, band 4).

(A)

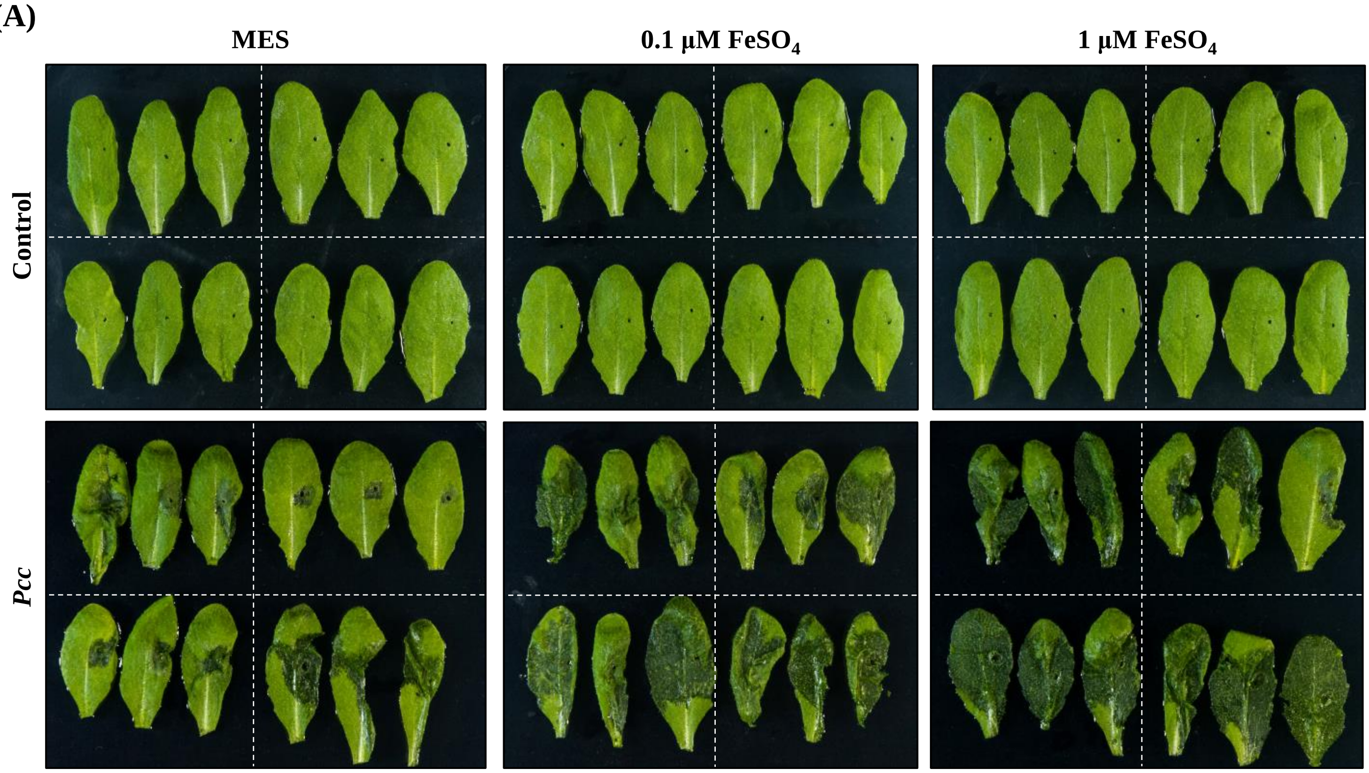

Ev	OE
ΔSP	RNAi

(B)

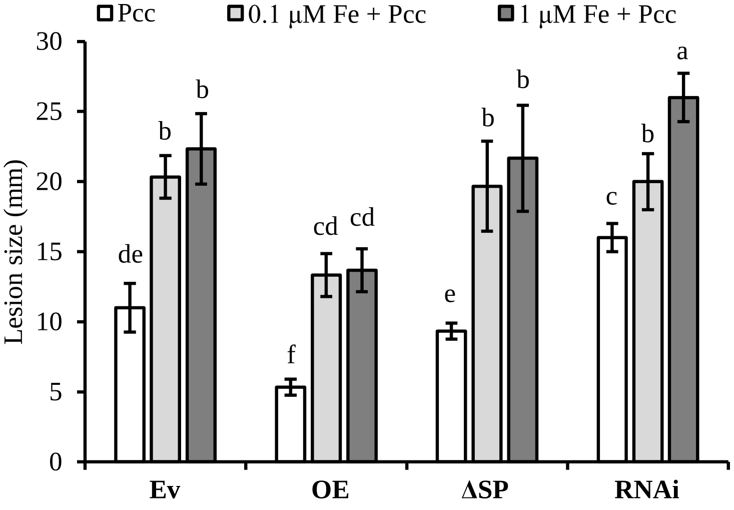

Figure S5. Infiltration of iron significantly increases *Pcc*-mediated symptoms in all transgenic plants. (**A**) Transgenic *Arabidopsis thaliana* plants were infiltrated with/without $FeSO_4$, followed by inoculation with *Pcc* or water (control), and *Pcc*-mediated symptoms were evaluated at 20 hpi. (**B**) The maximal lesion sizes (diameter; mm) on leaves, denoting bacterial soft rot severity, were determined at 20 hpi. Values are means ± standard errors from eight samples for each line and each treatment in a single experiment that was repeated at least three times with similar results. Different letters above each bar indicate significant difference (LSD post hoc two-way ANOVA, $P < 0.05$).

(A)

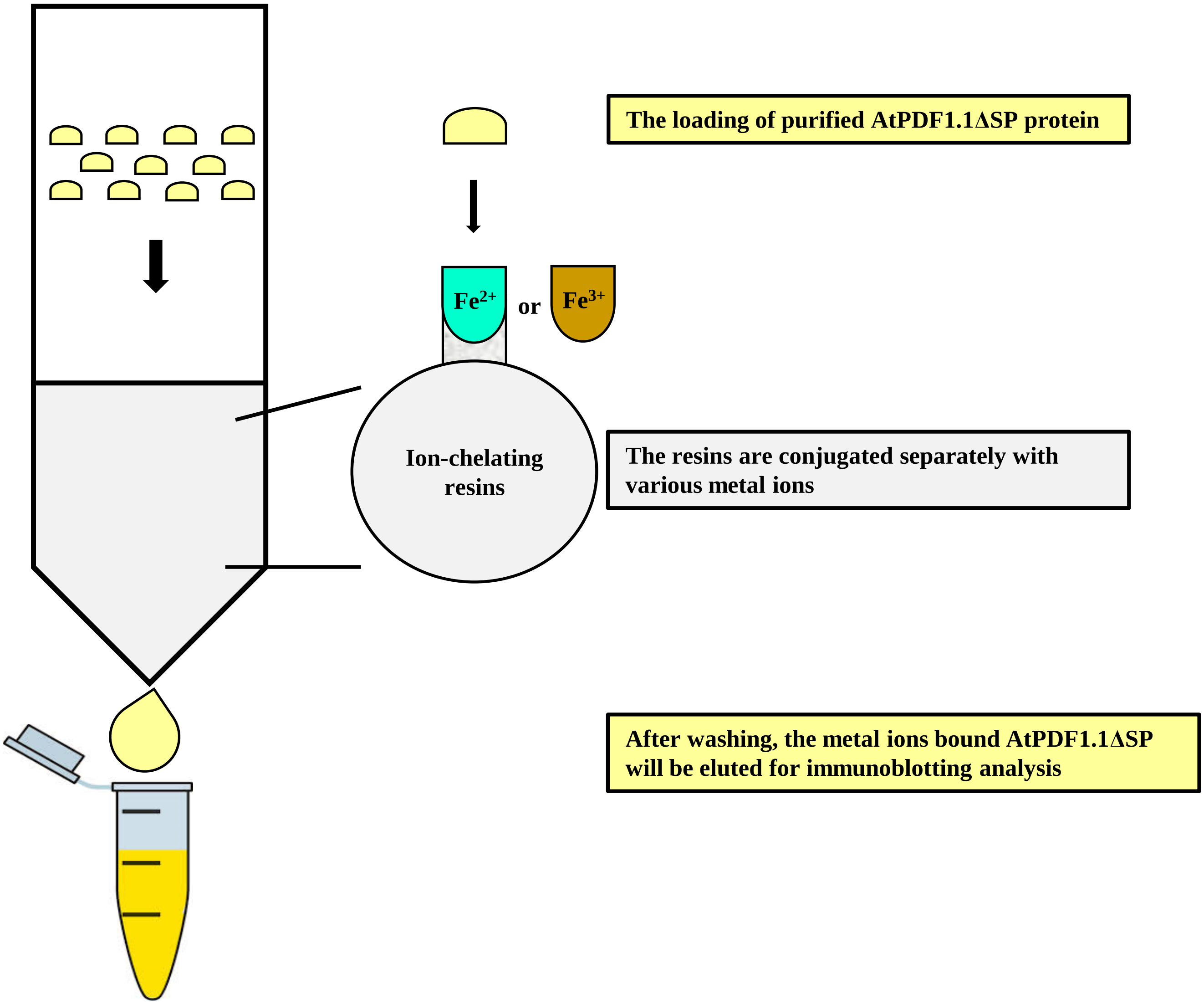

(B)

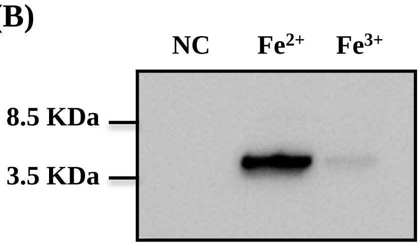

Figure S6. AtPDF1.1 protein potentially binds to iron ions. (**A**) Schematic diagram of AtPDF1.1 binding affinity. Toyopearl AF-Chelate-650 resins were packaged in a column and conjugated separately with metal ions. AtPDF1.1ΔSP recombinant protein (5 μg) was loaded into the metal ion conjugated resins column to allow binding. After washing, the metal bound AtPDF1.1 was eluted. (**B**) The eluted AtPDF1.1 protein was subjected to immunoblotting analysis using anti-AtPDF1.1 antibody. AtPDF1.1ΔSP recombinant protein without metal ion treatment was used as a negative control (NC). The experiment was repeated at least three times with similar results.

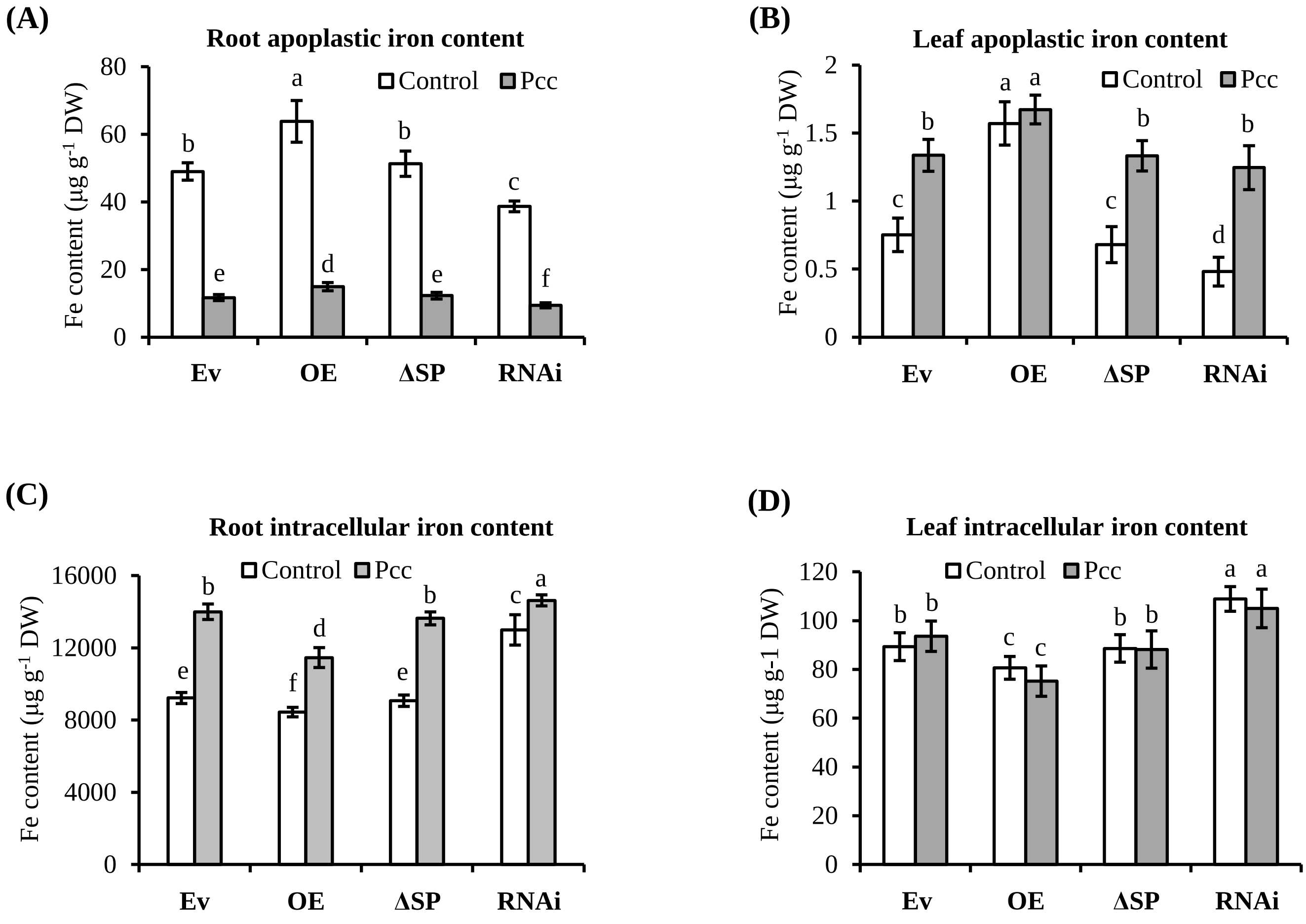

Figure S7. AtPDF1.1 protein tends to accumulate irons in the apoplast. Hydroponically grown transgenic plants were inoculated with *Pcc* or water (control). Roots (**A**) and systemic leaves (B) were harvested at 24 hpi, and the apoplastic iron content was measured. Values are means ± standard errors from six samples for each line and each infection in a single experiment that was repeated at least three times with similar results. The intracellular iron content of roots (**C**) and systemic leaves (**D**) was also measured. Values are means ± standard errors from eighteen samples for each line and each infection in a single experiment that was repeated at least three times with similar results. Different letters above each bar indicate significant difference (LSD post hoc two-way ANOVA, $P < 0.05$).

(A)

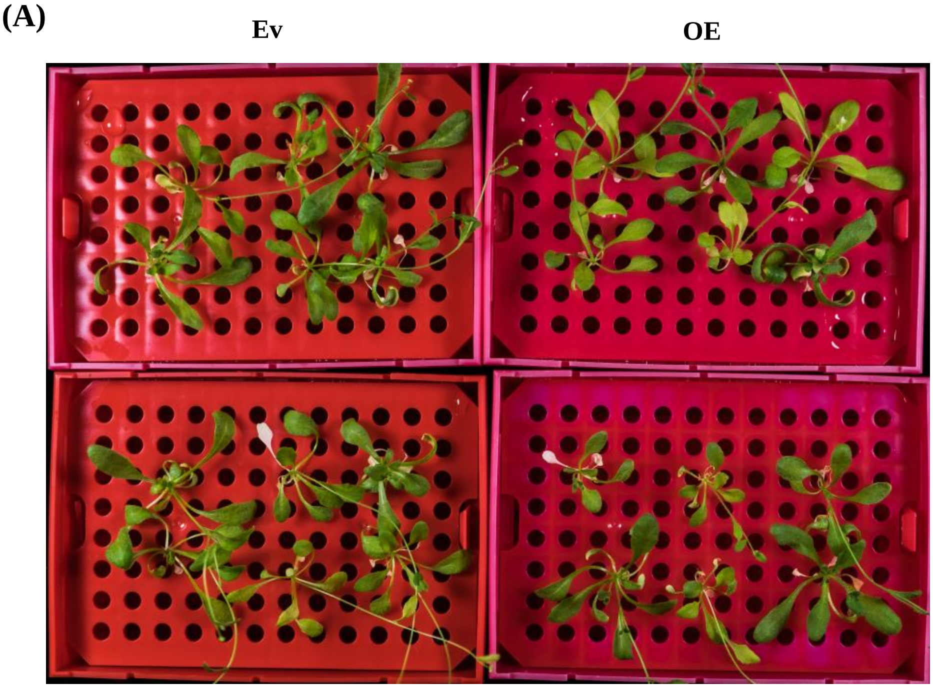

(B)

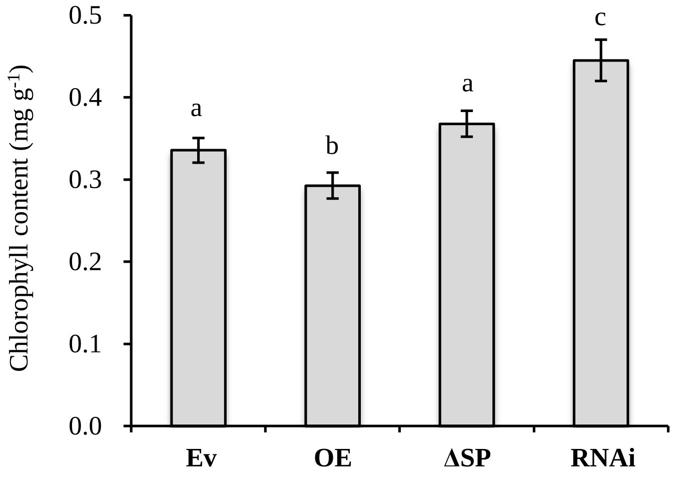

Figure S8. ***AtPDF1.1*** **OE plants had less chlorophyll content than the other transgenic plants.** (**A**) Transgenic *Arabidopsis* plants grown in ½ MS hydroponic medium. (**B**) The chlorophyll content of the transgenic plants described in panel (**A**) was measured. Values are means ± standard errors from six samples for each line in a single experiment that was repeated at least three times with similar results. Different letters above each bar indicate significant difference (LSD post hoc one-way ANOVA, $P < 0.05$).

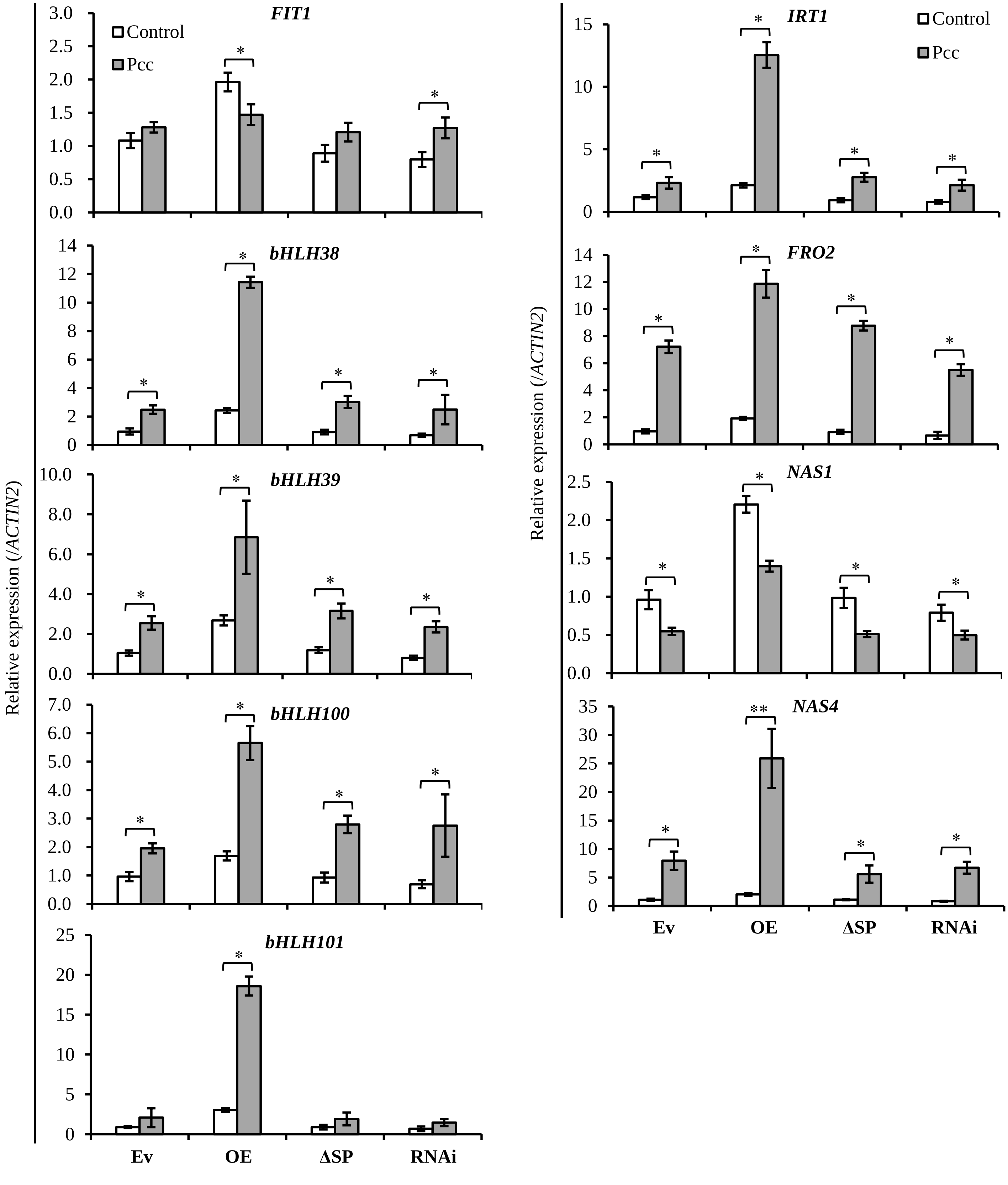

Figure S9. Iron deficiency-associated genes are increased after infection of *Pcc*. Hydroponically grown transgenic *A. thaliana* plants were inoculated with *Pcc* or water (Control). The systemic leaves and roots were harvested at 24 hpi, and the expression levels of genes associated with iron homeostasis (*FIT1*, *bHLH38*, *bHLH39*, *bHLH100 and bHLH101*) in the leaves, and iron deficiency response (*IRT1*, *FRO2*, *NAS1 and NAS4*) in the roots were monitored by qPCR and compared with expression in the empty vector control plant treated with water (0 h, defined value of 1). Transcript levels were normalized to those of *ACTIN2*. Values are means ± standard errors from twelve samples for each line and each infection in a single experiment that was repeated at least three times with similar results. Asterisk (*) above each bar indicate significant difference (LSD post hoc two-way ANOVA, * $P < 0.05$, ** $P < 0.01$).

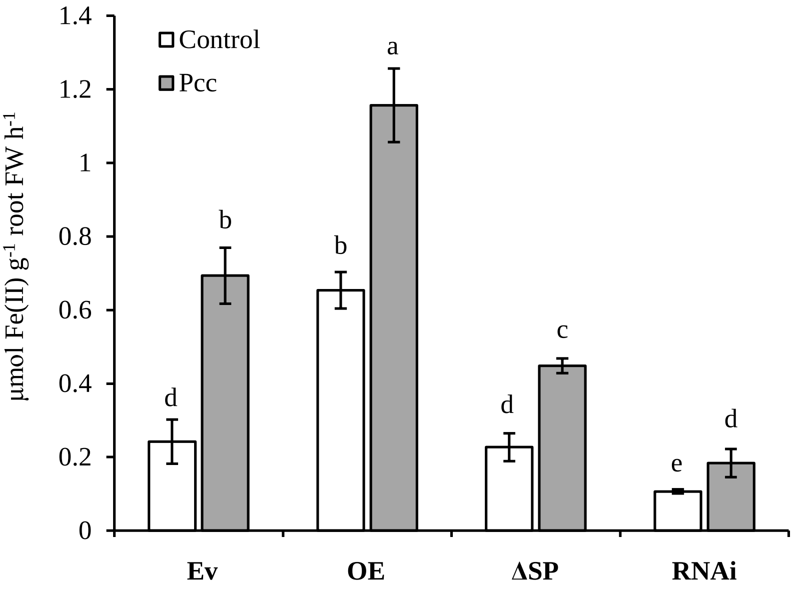

Figure S10. Ferric chelate reductase (FCR) activities are enhanced with expression of *AtPDF1.1* and infection of *Pcc*. Hydroponically grown transgenic *Arabidopsis* plants were inoculated with *Pcc* or water (control). The roots were harvested at 24 hpi and FCR activity assay was performed. Values are means ± standard errors from twelve samples for each line and each infection in a single experiment that was repeated at least three times with similar results. Different letters above each bar indicate significant difference (LSD post hoc two-way ANOVA, $P < 0.05$).

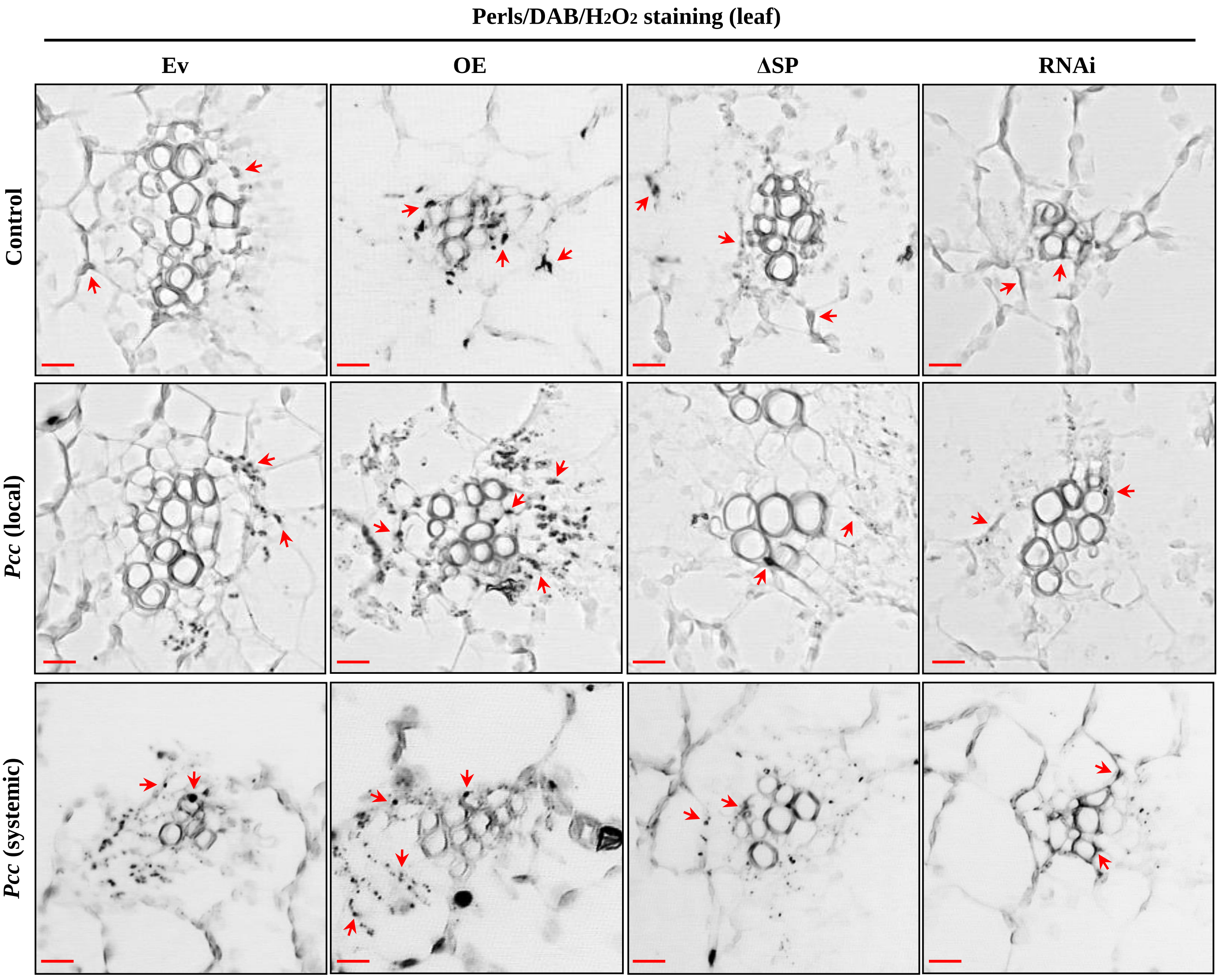

Figure S11. ***Pcc*** **infection induced apoplastic iron accumulation both locally and systemically.** Transgenic plants were inoculated with *Pcc* or water (control, upper panel), and the local leaves (middle panel) and systemic leaves (lower panel) were harvested at 4 or 24 hpi, respectively. Leaves were subjected to Perls/DAB/H_2O_2 staining and examined. Each line was repeated at least three times with similar results. The arrows indicate iron accumulating in the apoplast. Transverse section. Bars, 10 µm.

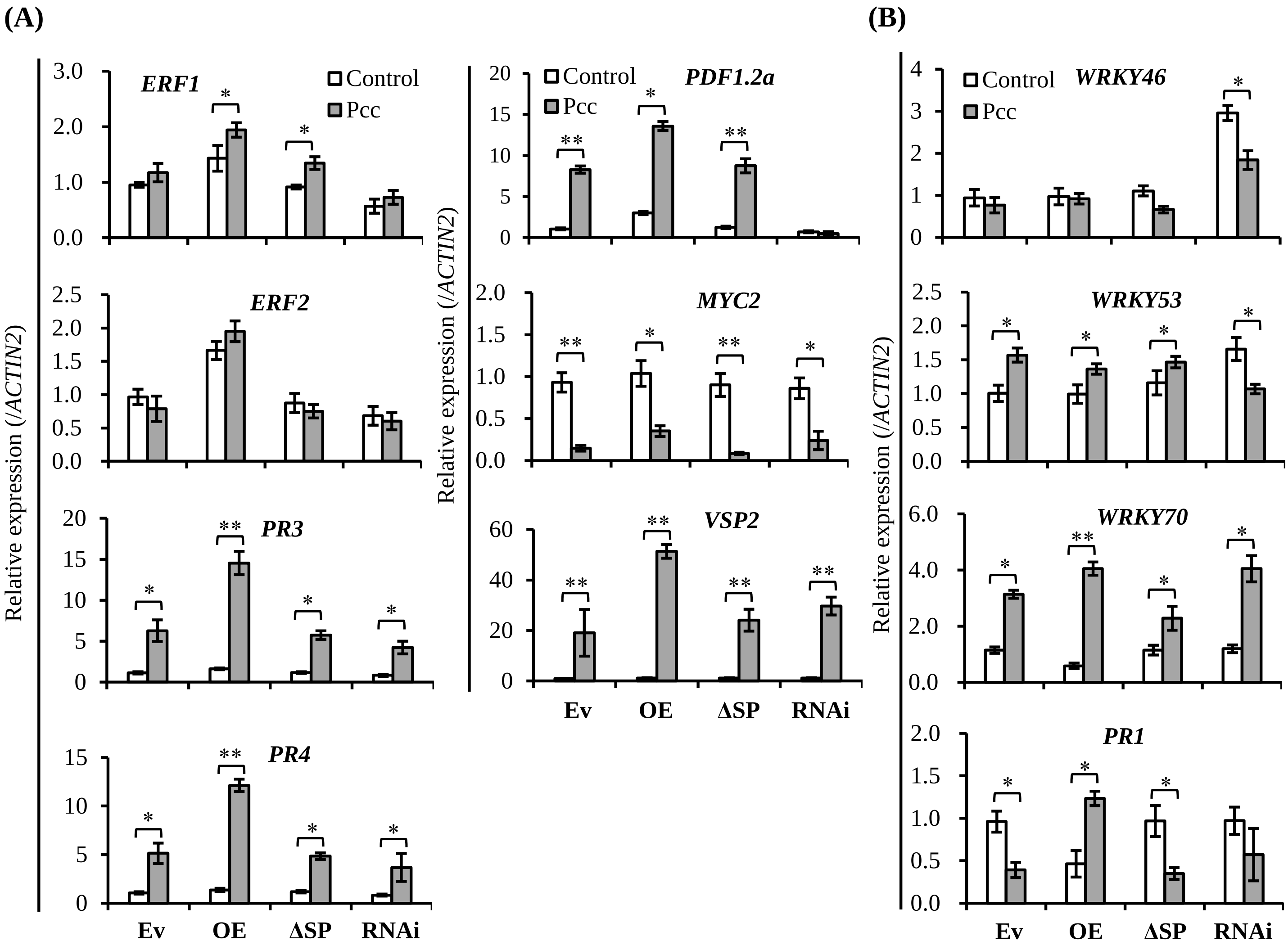

Figure S12. Downstream genes of *ERF1/2* are activated by *Pcc* infection. Hydroponically grown transgenic *A. thaliana* plants were inoculated with *Pcc* or water (control). The systemic leaves were harvested at 24 hpi and the JA/ET-responsive genes (*ERF1*, *ERF2*, *PR3*, *PR4*, *PDF1.2a*, *MYC2* and *VSP2*; **A**) and SA-responsive genes (*WRKY46*, *WRKY53*, *WRKY70* and and *PR1*; **B**) were monitored by qPCR and compared with expression in empty vector control plants treated with water (0 h, defined value of 1). Transcript levels were normalized to those of *ACTIN2*. Values are means ± standard errors from twelve samples for each line and each infection in a single experiment that was repeated at least three times with similar results. Asterisk (*) above each bar indicate significant difference (LSD post hoc two-way ANOVA, * $P < 0.05$, ** $P < 0.01$).

(A)

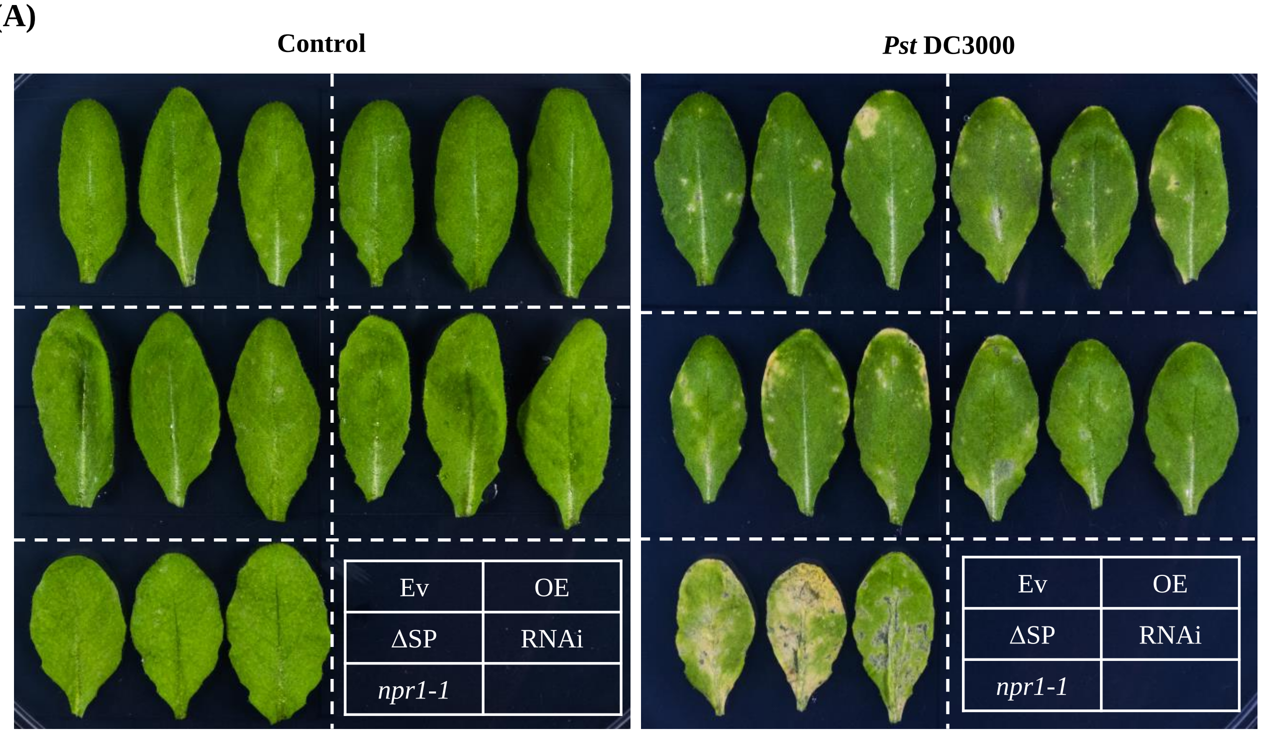

(B)

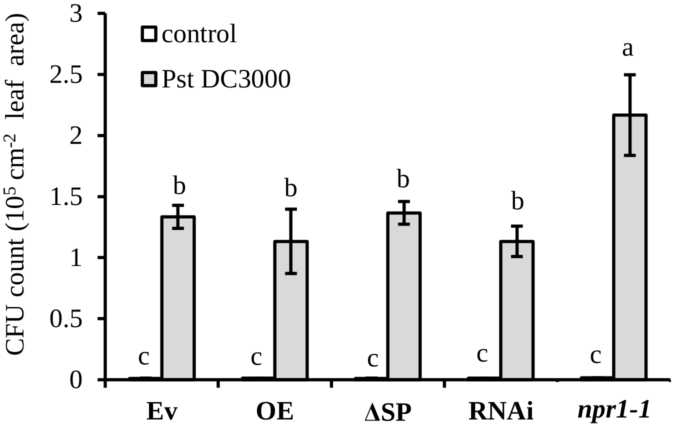

Figure S13. *AtPDF1.1* transgenic plants do not confer protection against *P. syringae* pv. *tomato* DC3000. Transgenic plants and *npr1-1* mutant were inoculated with a liquid bacteria culture of *Pst* DC3000 or 10 mM $MgSO_4$ (control). The symptoms (**A**) and bacterial counts per area (**B**) were evaluated at 3 days after infection. Values are means ± standard errors from twelve samples for each line and each infection in a single experiment that was repeated at least three times with similar results. Different letters above each bar indicate significant difference (LSD post hoc two-way ANOVA, $P < 0.05$).

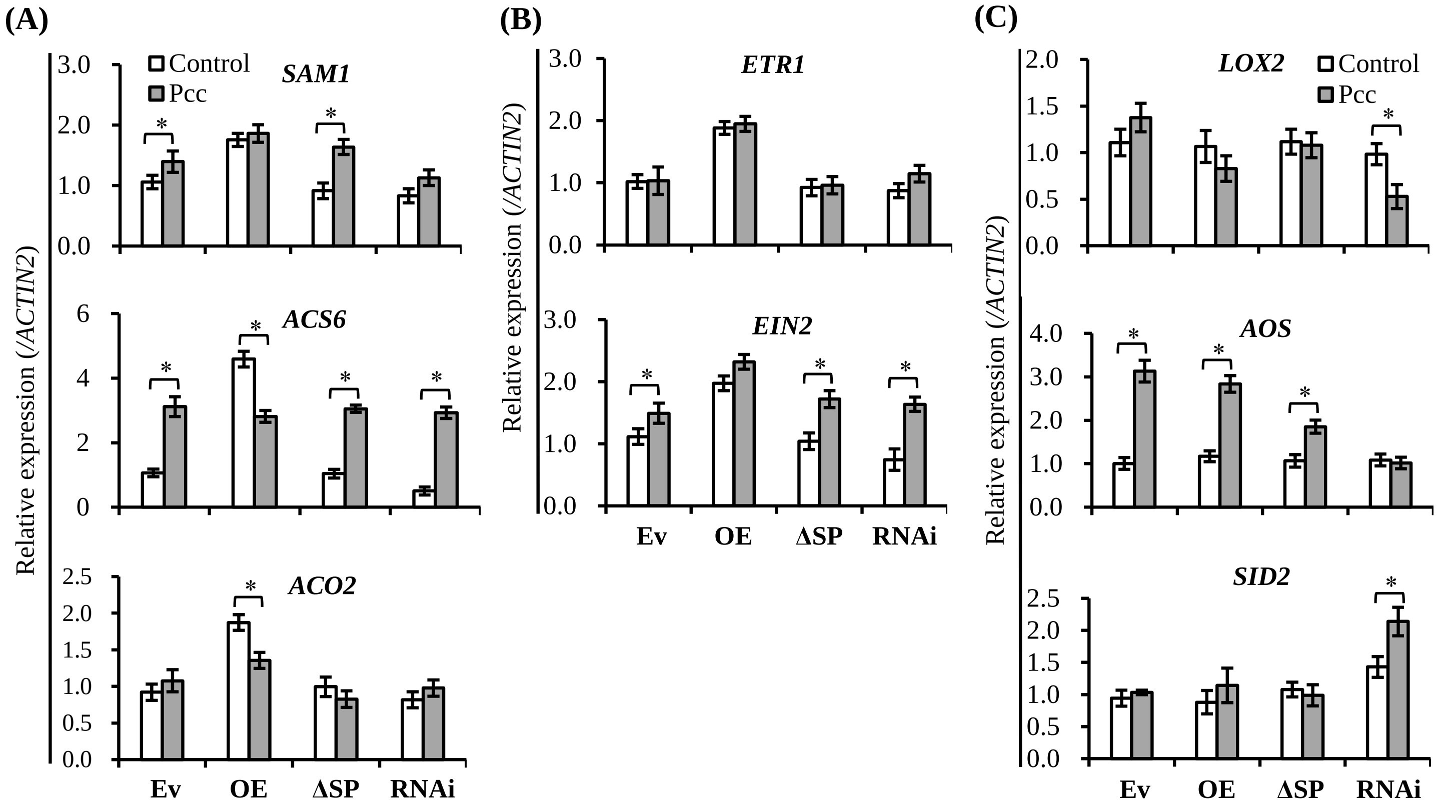

Figure S14. ET-biosynthesis and signalling genes are up-regulated by infection of *Pcc*. Hydroponically grown transgenic *A. thaliana* plants were inoculated with *Pcc* or water (control). The roots were harvested at 24 hpi and transcript levels of ET-biosynthesis genes (*SAM1*, *ACS6* and *ACO2*; **A**), ET-signalling genes (*ETR1* and *EIN2*; **B**) and JA-/SA-biosynthesis genes (*LOX2*, *AOS* and *SID2*; **C**) were monitored by qPCR and compared with expression in empty vector control plants treated with water (0 h, defined value of 1). Transcript levels were normalized to *ACTIN2*. Values are means ± standard errors from twelve samples for each line and each infection in a single experiment that was repeated at least three times with similar results. Asterisks (*) above each bar indicate significant difference (LSD post hoc two-way ANOVA, * $P < 0.05$).

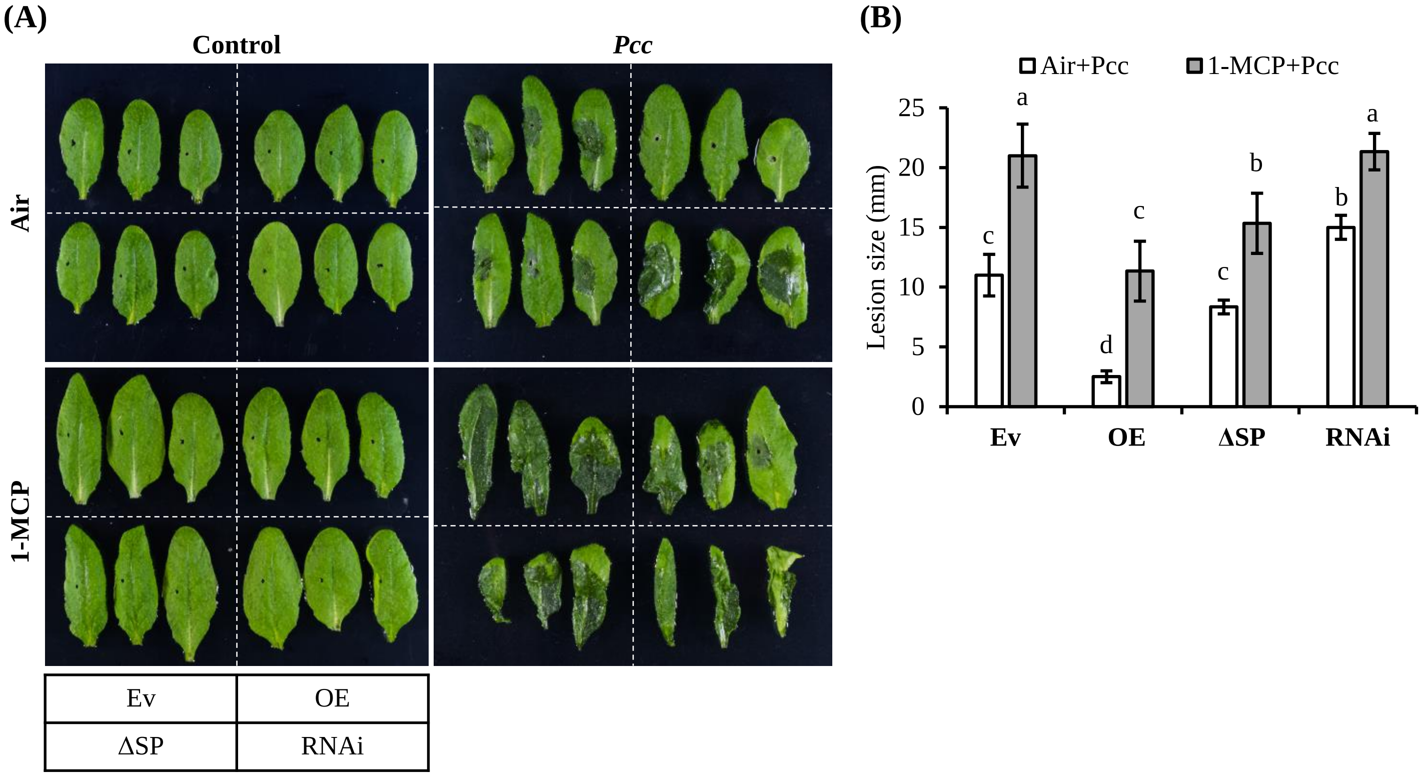

Figure S15. Application of ethylene inhibitor significantly enhances the severity of *Pcc*-mediated disease. (**A**) Transgenic *Arabidopsis thaliana* plants were treated with/without 1-MCP, followed by inoculation with *Pcc* or water (control), and *Pcc*-mediated symptoms were evaluated at 20 hpi. (**B**) The maximal lesion sizes (diameter; mm) on leaves, denoting bacterial soft rot severity, were determined at 20 hpi. Values are means ± standard errors from eight samples for each line and each treatment in a single experiment that was repeated at least three times with similar results. Different letters above each bar indicate significant difference (LSD post hoc two-way ANOVA, $P < 0.05$). Revised

Table S1. List of primers used for vector construction.

Primer name	Sequence 5'->3'
AtPDF1.1-F	AAAAA GCAGG CTTCA TGGCT AAGTC TGCTA CCAT
AtPDF1.1-R	AGAAA GCTGG GTATT AACAT GGGAA GTAGC AGATA CA
AtPDF1.1-R (Δstop)	AGAAA GCTGG GTATT CACAT GGGAA GTAGC AGATA CA
AtPDF1.1-F (ΔSP)	AAAAA GCAGG CTCAA TGCAG AAGTT GTGCG AGAGG CC
AtPDF1.1 RNAi-F	AAAAA GCAGG CTTGG TGGAA GCACA GAAGT TG
AtPDF1.1 RNAi-R	AGAAA GCTGG GTTCA AGGTT AATGC ACTGA TT
AtPDF1.1(SP)-R	AGAAA GCTGG GTTCT GTGCT TCCAC CACCA TCGGT
AtPDF1.1pro-SacI-F	GAGCTC ATGCT AATCC CATAA TTGAT CCCTT
AtPDF1.1pro-SpeI-R	ACTAGT GACTATTGTTTATACTTAGTTGTTTTCG
attB1-adapter	GGGGA CAAGT TTGTA CAAAA AAGCA GGCT
attB2-adapter	GGGGA CCACT TTGTA CAAGA AAGCT GGGT

Table S2. List of primers used to quantify gene expression levels.

Gene name	Forward primer (5'->3')	Reverse primer (5'->3')
AtPDF1.1	GGACATGGTCCGGAGTTT	CAAGATCCATGTCGTGCTTTC
AtPDF1.2a	CCATCATCACCCTTATCTT	CTGGGAAGACATAGTTGCAT
AtPDF1.3	ATGGCTAAGTCTGCT	TGCAAGATCCATGTTTTGCC
FIT1	CAGTCACAAGCGAAGAAACTCA	CTTGTAAAGAGATGGAGCAACACC
HLH38	TACCGACGCAAGAAGATCAAC	TCCTTGAGATCTTGATGATGAAAC
bHLH39	GACTTATGGAGCTGTTACAGCGGT	CTTCAAGCTTCGAGAAACCGTCGCA
bHLH100	TGTCCCTCCATTATATCCCAATTT	CGTCGTCGGTTTCATAGAAG CT
bHLH101	TTCATTCCTTTTCACCTACTCTCC	TATTGAGTCCATCATTGCTTTGTT
IRT1	CACCATTCGGAATAGCGTTA GG	CCAGCGGAGCATGCATTTA
FRO2	TGCCGCACATGACGAATCT	CTTAGCGCCGCTGATTCC
NAS1	GGCTTTCAACTCTTGACCATCTAT	ACAAGACATGAAATGAAAAGAGCA
NAS4	TGTAATCTCAAGGAAGCTAGGTG	CAGTTACACGCGAGATCCG
LOX2	ATGCTACGTCATGCTGGCTATGGA	TGCCGCTATTATGTATGGCTCCGT
AOS	ACGACGCGGCGTTTAAAGTCAAAG	ACGAATCTCTCCGGCACAAACTCA
SID2	TTCTGGGCTCAAACACTAAAAC	GGCGTCTTGAAATCTCCATC
ERF1	TTCCCTTCAACGAGAACGAC	TAGGTTTGTTGCGTGGACTG
ERF2	TGTTTCACAGAGAGTTGGGGAGGT	GAGGAGTCCGTACACCAACATGTC
MYC2	AGCAACGTTTACAAGCTTTGATTG	TCATACGACGGTTGCCAGAA
PR1	ACACCTCACTTTGGCACATC	GAGTGTGGAAAACGCAAAGA
PR3	GTATGGCTGGACCGCCTTC	GTTCTTCACCCTTAAACACTTGC
PR4	GGCAGCCTGATTCCAGAAA	CCCTCGACAATACATATATAGC
VSP2	CGCCAAATTCTAGTTAAGCACACA	TCGATTGGTGCAACAAATGCT
WRKY46	AGCAAGTTCAGAAATCAGACACAG	CCGATTGCTCTGTAACATGTACTC
WRKY53	GACGGGGATGCTACGGTTT	TTTTGGGTAATGGCTGGTTTG
WRKY70	GAGGACGCATTTTCTTGGAG	TTGCTCTTGGGAGTTTCTGC
SAM1	CTCGTCTCAAACCGTTCCTT	GCGATCAGATCTCGAAGAAGAG
ACS6	TTAGCTAATCCCGGCGATGG	ACAAGATTCACTCCGGTTCTCCA
ACO2	TCTACGTTCGTCACCTCCCTCA	CTCTTACCAAAGTCTTTCATGGCC
ETR1	CTCCTTCTCCGTCGCTCTC	CCTCTCTCACACATACACACAC
EIN2	CGTTCTCAACCGCCTACAG	CGGACTCGCTCTCTGGTG
UBC21	TTCAA ATGGA CCGCT CTTAT CA	AAACACCGCCTTCGTAAGGA
ACT2	TTTTGCGTTTTAGTCCCATTG	GGCATCAATTCGATCACTCA
AhPDF1.1b	GCAGAAATCAGTGCATTAGACTTG	TAAAATACACACACACGAAGCAC
AhPDF1.2b	AGTTTTGCGAGAAGCCAAGTGGT	GTAAAATACACACGACACAGA
AhPDF1.4	AGATGATGGCGGTGGAAGCAAG	AAACCCGGGAACTGAGCGTG
Ahactin	GGTAA CATTG TGCTC AGTGG TGG	AACGA CCTTA ATCTTCATGCTGC

Method S1. Plant materials and growth conditions.

All *A. thaliana* genotypes used in this study were in the Col-0 background and *A. halleri* was kindly provided by Dr. Kuo-Chen Yeh[71]. Seeds were surface sterilized with 1% sodium hypochlorite for 20 min and then washed ten times with sterilized water. For vernalisation, disinfected seeds were imbibed at 4°C for 3 days in the dark before sowing onto half-strength Murashige and Skoog (½ MS) (Duchefa Biochemie [M0221]) agar containing 1% sucrose, 0.5% agar gel (Sigma (A3301)) and 0.5 g L^{-1} MES (J.T.Baker (4014-02)) and adjusted to pH 5.6 using 1 M KOH. Plates were incubated at 23°C under long day conditions [16/8 h (light/dark)] and a light intensity of 120 µmol $m^{-2}s^{-1}$. For selection of transgenic lines, seeds were plated onto ½ MS agar supplemented with 15 µg ml^{-1} hygromycin B (MDBio). Seeds that germinated and survived for an additional five days were transferred to ½ MS plates without hygromycin and grown for another three days, and then grown hydroponically or in soil. For hydroponic cultures, the seedlings were grown on ½ MS liquid medium containing 0.5% sucrose and 10 mg L^{-1} Meropenem (China Chemical & Pharmaceutical) for three weeks with the medium being changed weekly.

Method S2. Generation of transgenic plants.

To generate *AtPDF1.1 OE* (OE) and *AtPDF1.1ΔSP* (ΔSP) transgenic plants, the full-length *AtPDF1.1*-encoding sequence, with or without the predicted signal peptide (SP) sequence, respectively, were amplified from *A. thaliana* Col-0 by PCR using the AtPDF1.1-F/AtPDF1.1-R and AtPDF1.1-F (ΔSP)/AtPDF1.1-R primer pairs (Table S1). To generate *AtPDF1.1 RNAi* transgenic plants, a partial fragment of *AtPDF1.1* was amplified using the AtPDF1.1RNAi-F/AtPDF1.1 RNAi-R primer pair (Table S1). The amplified products were cloned into the pDONR221 vector (Invitrogen) using the Gateway BP reaction according to the manufacturer's instructions. The fragments were verified by sequencing and the DNA fragments recombined into the destination vector pH2GW7 or pH7GWIWG2(I) using the Gateway LR reaction[72]. To generate *AtPDF1.1:GFP*, *AtPDF1.1ΔSP:GFP* and *AtPDF1.1 (SP only):GFP* transgenic plants used in the cellular localization assay, gene fragments were amplified from *A. thaliana* Col-0 using the AtPDF1.1-F/AtPDF1.1-R (Δstop), AtPDF1.1-F (ΔSP)/AtPDF1.1-R (Δstop) and AtPDF1.1-F/AtPDF1.1(SP)-R primer pairs, respectively (Table S1). The fragments were verified by sequencing and recombined into the destination vector pH7FWG2 using the Gateway cloning system, as above[72]. The constructs were transformed into *A. thaliana* by the floral dip method using *Agrobacterium tumefaciens* strain GV3101 (pMP90)[73]. The pH2GW7 and pH7FWG2 empty vectors were also transformed into *A. thaliana* to generate *Ev* and *Ev-GFP* control transgenic plants. For the *AtPDF1.1* promoter study, the promoter region (1.2 kb from the start codon) of the *AtPDF1.1*-encoding gene[58] was amplified using the AtPDF1.1pro-SacI-F/AtPDF1.1pro-SpeI-R primer pair (Table S1). The cauliflower mosaic virus (CaMV) 35S promoter region of the *AtPDF1.1*/pH7FWG2 construct (*AtPDF1.1:GFP*) was replaced by the *AtPDF1.1* promoter region via ligation into the *Sac*I and *Spe*I-restriction endonuclease sites.

Method S3. Immunoblot analysis.

Total proteins from transgenic plants were extracted using RIPA Buffer (SIGMA, [R0278]), as described by the supplier, and adjusted to the same concentration with SDS sample buffer. Proteins were fractionated by electrophoresis on 12% Tris SDS–PAGE gels and equal loading validated using Coomassie blue staining (Thermo Scientific [20278]), then another PAGE with the same loading was transferred onto a PVDF membrane (GE Healthcare, [10600021]) for immunoblot analysis[18]. The membrane was probed with primary anti-GFP antibody (GFP(FL); Santa Cruz Biotechnology, [sc-8334]) at a dilution of 1:1000 for 1 hour at room temperature, anti-EF1α antibody (Agrisera, [AS11 1633]) at a dilution of 1:1000 overnight at 4 degrees or anti-EF1B-α2 antibody (Agrisera, [AS10 679]) at a dilution of 1:2000 for 2 hours at room temperature, followed by secondary HRP-rec-Protein G antibody (Invitrogen, [10-1223]) at a dilution of 1:1000 for 1 hour at room temperature. The antisera were washed in TBS buffer (OmicsBio [IB3052]) containing 0.05% Tween 20 (J.T.Baker, [X251-07]) four times for 40 min. The AtPDF1.1ΔSP recombinant proteins were fractionated by electrophoresis on 16.5% Tricine-SDS-PAGE gels[74] and immunoblot analysis was performed as above using an AtPDF1.1 antibody (epitope, NLEKARHGSC) at a dilution of 1:1000 for 1 hour at room temperature and HRP-rec-Protein G antibody as primary and secondary antibody, respectively. Horseradish peroxidase (HRP) activity was detected using the Western Lighting *Plus-ECL* substrate (PerkinElmer, [PK-NEL105]).

Method S4. Metal binding assay.

The *AtPDF1.1ΔSP*-encoding fragment was amplified from *A. thaliana* cDNA using the AtPDF1.1-F (ΔSP)/AtPDF1.1-R primer pair, and the sequence was verified and cloned into the pET-53-DEST destination vector (Novagen) using the Gateway cloning system (Invitrogen), as described above. The construct was transformed into Rosseta-gami B (DE3) pLySs *Escherichia coli* cells (Novagen) to generate AtPDF1.1ΔSP recombinant protein. The N-terminal His-tag was cleaved using cyanogen bromide (CNBr), as described by Marques[75], and native-oxidative folding was performed using the protocol described by Meindre[76]. Toyopearl AF-Chelate-650 resin (Tosoh Bioscience) derivatized from iminodiacetic acid (IDA), was conjugated separately with iron ions, including $FeSO_4$ (Fe^{2+}), $Fe_2(SO_4)_3 \cdot xH2O$ (Fe^{3+}) and 100 mM Tris-HCl buffer, pH 8.5 (negative control) and packaged into columns (Fig. S6). For the affinity assays, AtPDF1.1ΔSP recombinant protein was allowed to pass through the metal ion-conjugated columns. Proteins that bound to the metal ion-conjugated resins were washed with wash buffer (60 mM imidazole, 0.5 M NaCl, 10 mM Tris-Cl, pH7.9) and eluted with elution buffer (1 M imidazole, 0.5 M NaCl, 20 mM Tris-Cl, pH6) followed by nickel affinity purification as described by the supplier (Tosoh Bioscience) and subjected to immunoblot analysis using anti-AtPDF1.1 antibody described as above.

Method S5. Measurement of metal concentration.

For intracellular iron content quantitation, leaves and roots from four-week-old hydroponically-grown transgenic plants were thoroughly rinsed with 0.5 mM $CaSO_4$ solution, followed by incubating in apoplastic Fe removal solution (1.5 mM 2,20-Bipyridyl (BIP), 0.5 mM $CaSO_4$, and 5 mM sodium dithionite) under continuous N_2 bubbling through the solution for 10 min[77]. After three brief Milli-Q H_2O washing, the samples were dried at 70°C for 3 days and dry weight was recorded. Iron content was measured using Inductively Coupled Plasma-Optic Emission Spectroscopy (ICP-OES; OPTIMA 5300; Perkin-Elmer), as described by Shanmugam[78]. For the apoplastic iron assay, leaves and roots from hydroponically-grown transgenic plants were harvested and washed 5 times with 0.5 mM $CaCl_2$ solution (pH 5.6) for 1 min. The root and leaf apoplastic iron levels were measured as previously described[79,80].

Method S6. Accession numbers.

The genes used in this article are: *AtPDF1.1*, AT1G75830; *AtPDF1.2a*, AT5G44420; *AtPDF1.3*, AT2g26010; *FIT1*, AT2G28160; *bHLH38*, AT3G56970; bHLH39, AT3G56980; bHLH100, AT2G41240; *bHLH101*, AT5G04150; *IRT1*, AT4G19690; *FRO2*, AT1G015800; *NAS1*, AT5G04950; *NAS4*, AT1G56430; *LOX2*, AT3G45140; *AOS*, AT5G42650; *SID2*, AT1G74710; *ERF1*, AT3G23240; *ERF2*, AT5G47220; *PR3*, AT3G12500; *PR1*, AT2G14610; *PR4*, AT3G04720; *MYC2*, AT1G32640; *VSP2*, AT5G24770; *WRKY46*, AT2G46400; *WRKY53*, AT4G23810; *WRKY70*, AT3G56400; *SAM1*, AT1G02500; *ACS6*, AT4G11280; *ACO2*, AT1G62380; *ETR1*, AT1G66340; *EIN2*, AT5g03280; *UBC21*, AT5G25760; *ACTIN2*, AT3G18780. UniProtKB accession number of AtPDF1.1 is P30224.

Supplementary References

71 Tsednee, M., Yang, S. C., Lee, D. C. & Yeh, K. C. Root-secreted nicotianamine from *Arabidopsis halleri* facilitates zinc hypertolerance by regulating zinc bioavailability. *Plant Physiol.* **166**, 839-852 (2014).

72 Karimi, M., Inze, D. & Depicker, A. GATEWAY vectors for Agrobacterium- mediated plant transformation. *Trends Plant Sci.* **7**, 193-195 (2002).

73 Sanjaya *et al.* Overexpression of *Arabidopsis thaliana tryptophan synthase beta 1* (*AtTSB1*) in *Arabidopsis* and tomato confers tolerance to cadmium stress. *Plant Cell Environ.* **31**, 1074-1085 (2008).

74 Schagger, H. Tricine-SDS-PAGE. *Nat. Protoc.* **1**, 16-22 (2006).

75 Marques, L., Oomen, R. J., Aumelas, A., Le Jean, M. & Berthomieu, P. Production of an *Arabidopsis halleri* foliar defensin in *Escherichia coli*. *J. Appl. Microbiol.* **106**, 1640-1648 (2009).

76 Meindre, F. *et al.* The nuclear magnetic resonance solution structure of the synthetic AhPDF1.1b plant defensin evidences the structural feature within the gamma-motif. *Biochemistry* **53**, 7745-7754 (2014).

77 Kovacs, K. *et al.* Revisiting the iron pools in cucumber roots: identification and localization. *Planta* **244**, 167-179 (2016).

78 Shanmugam, V., Wang, Y. W., Tsednee, M., Karunakaran, K. & Yeh, K. C. Glutathione plays an essential role in nitric oxide-mediated iron-deficiency signalling and iron-deficiency tolerance in *Arabidopsis*. *Plant J.* **84**, 464-477 (2015).

79 Nikolic, M. & Römheld, V. Does high bicarbonate supply to roots change availability of iron in the leaf apoplast? *Plant Soil* **241**, 67-74 (2002).

80 Masalha, J., Kosegarten, H., Elmaci, Ö. & Mengel, K. The central role of microbial activity for iron acquisition in maize and sunflower. *Biol. Fert. Soils* **30**, 433-439 (2000).
